# Pathogenic seedborne viruses are rare but *Phaseolus vulgaris* endornaviruses are common in bean varieties grown in Nicaragua and Tanzania

**DOI:** 10.1371/journal.pone.0178242

**Published:** 2017-05-25

**Authors:** Noora Nordenstedt, Delfia Marcenaro, Daudi Chilagane, Beatrice Mwaipopo, Minna-Liisa Rajamäki, Susan Nchimbi-Msolla, Paul J. R. Njau, Deusdedith R. Mbanzibwa, Jari P. T. Valkonen

**Affiliations:** 1 Department of Agricultural Sciences, University of Helsinki, Helsinki, Finland; 2 Nicaraguan Institute of Agricultural Technology (CNIAB-INTA), Managua, Nicaragua; 3 Sokoine University of Agriculture, Morogoro, Tanzania; 4 Mikocheni Agricultural Research Institute, Dar es Salaam, Tanzania; Oklahoma State University, UNITED STATES

## Abstract

Common bean (*Phaseolus vulgaris*) is an annual grain legume that was domesticated in Mesoamerica (Central America) and the Andes. It is currently grown widely also on other continents including Africa. We surveyed seedborne viruses in new common bean varieties introduced to Nicaragua (Central America) and in landraces and improved varieties grown in Tanzania (eastern Africa). Bean seeds, harvested from Nicaragua and Tanzania, were grown in insect-controlled greenhouse or screenhouse, respectively, to obtain leaf material for virus testing. Equal amounts of total RNA from different samples were pooled (30–36 samples per pool), and small RNAs were deep-sequenced (Illumina). Assembly of the reads (21–24 nt) to contiguous sequences and searches for homologous viral sequences in databases revealed *Phaseolus vulgaris endornavirus 1* (PvEV-1) and PvEV-2 in the bean varieties in Nicaragua and Tanzania. These viruses are not known to cause symptoms in common bean and are considered non-pathogenic. The small-RNA reads from each pool of samples were mapped to the previously characterized complete PvEV-1 and PvEV-2 sequences (genome lengths ca. 14 kb and 15 kb, respectively). Coverage of the viral genomes was 87.9–99.9%, depending on the pool. Coverage per nucleotide ranged from 5 to 471, confirming virus identification. PvEV-1 and PvEV-2 are known to occur in *Phaseolus* spp. in Central America, but there is little previous information about their occurrence in Nicaragua, and no information about occurrence in Africa. Aside from *Cowpea mild mosaic virus* detected in bean plants grown from been seeds harvested from one region in Tanzania, no other pathogenic seedborne viruses were detected. The low incidence of infections caused by pathogenic viruses transmitted via bean seeds may be attributable to new, virus-resistant CB varieties released by breeding programs in Nicaragua and Tanzania.

## Introduction

Common bean (*Phaseolus vulgaris* L.; *Fabaceae*; referred to as CB here) is an annual legume that was domesticated independently in Mesoamerica (Central America) and the Andes over 7000 years ago [1]. Today CB is grown worldwide and is a vital source of nutrition in many developing countries. In Nicaragua (Central America) and Tanzania (eastern Africa), for example, CB is the second most important source of dietary protein and starch after maize [2,3,4,5]. However, in both countries the yields of CB are rather poor and can vary greatly owing to pests, diseases, weeds, weather and edaphic constraints. In this regard, better-yielding and well-adapted CB cultivars are being bred and introduced to improve yields [4,6,7,8].

Seedborne pathogens, including certain viruses, have great potential to reduce growth and yield of food crops because they interfere with plant growth from the beginning [9,10]. The seedborne viruses known to infect CB crops in Nicaragua include *Bean common mosaic virus* (BCMV, genus *Potyvirus; Potyviridae*) [11] and *Southern bean mosaic virus* (genus *Sobemovirus*) [12,13]. Other seedborne viruses of CB, such as *Bean common mosaic necrosis virus* (BCMNV; genus *Potyvirus*) [14,15] and *Cucumber mosaic virus* (CMV) [16], have been reported elsewhere in Mesoamerica. In Tanzania, BCMV and BCMNV occur in CB and forage legumes [17,18], and CB is also sometimes infected with *Cowpea mild mottle virus* (CPMMV; genus *Carlavirus*) [19]. Besides seeds, vectors such as aphids (e.g., BCMV, BCMNV, CMV), whiteflies (CPMMV) and leaf beetles (*Southern bean mosaic virus*) transmit these viruses [20–23]. Wild plants and weeds can act as virus reservoirs for transmission by vectors, as demonstrated by infection of wild legume species with BCMV and BCMNV in Tanzania and Uganda [17,22]. Even low seedborne transmission rates of viruses may be sufficient to cause severe disease epidemics when combined with efficient spread by vectors to susceptible crops [24].

CB can also be infected with *Phaseolus vulgaris endornavirus 1* (PvEV-1) and *Phaseolus vulgaris endornavirus 2* (PvEV-2). The complete sequences of PvEV-1 and PvEV-2 were characterized by Okada et al. [25] (NCBI sequence database accession nos. AB719397 and AB719398, respectively). The country of origin of these isolates is mentioned to be Brazil. We refer to them further on as PvEV-1-Okada and PvEV-2-Okada, respectively, in this study. There are also two other PvEV sequences deposited in NCBI, named PvEV-1-Brazil (KT456287) and PvEV-2-Brazil (KT456288) also characterized from the CB cultivar ‘Black Turtle Soup’. Their sequences are 16 and 3 nucleotides shorter, respectively, than those characterized by Okada et al. [25]. We refer to them as PvEV-1-Melo and PvEV-2-Melo according to the first author. We are not aware of publications reporting on these latter mentioned isolates.

Endornaviruses belong to the relatively newly established family *Endornaviridae* and include virus species infecting plants, fungi or oomycetes [26,27,28,29]. The genomes of endornaviruses consist of a single, single-stranded, linear, non-encapsidated RNA molecule of 9.8–17.6 kb depending on the virus. In the host, however, endornaviruses are typically found as double-stranded replicative intermediates [30]. Endornaviruses infecting plants contain a single open reading frame that encodes a large protein containing conserved domains for RNA helicase, glycosyltransferase and RNA-dependent RNA polymerase (RdRp). Vertical transmission of endornaviruses occurs at a high rate through seeds, pollen or fungal spores. Horizontal transmission by contact or vectors is not known to occur [31,32,33]. The impact, if any, of endornaviruses on their hosts remains elusive in most cases [25,34].

PvEV-1 has been reported in CB in Spain at high incidence [35]. Furthermore, PvEV-1 and PvEV-2 have been detected in a large number of CB accessions maintained in germplasm collections in the United States (USA) [36]. These accessions include CB landraces and improved cultivars grown in the aforementioned CB domestication centers in South and Central America. Nicaragua is located in one of the CB domestication centers, but little information is available concerning occurrence of PvEV-1 and PvEV-2 or the known pathogenic seedborne viruses in the CB varieties introduced to this country.

New, more virus-resistant CB varieties bred in USA, University of Samorano (Honduras), and International Center for Tropical Agriculture (CIAT) in Colombia have been released in Nicaragua during the past decade [7,8,37]. Before registration as cultivars, the new CB varieties introduced to Nicaragua are tested for adaptability and other important characteristics in La Compañia, a research station situated in Carazo—the Pacific region of Nicaragua. Also, the first seed generations of the new varieties are usually produced in La Compañia for distribution to farmers. Therefore, good phytosanitary status in the fields of La Compañia is of importance. The Tanzanian coast, in turn, is the area in eastern Africa where CB was first introduced by the Portuguese in the 16th century and spread from there to the inland areas by the Arab slave traders [3]. Today, the main CB growing areas in Tanzania are the highlands in the Arusha region (northern zone), the Great Lakes region (western zone), Southern Highlands, and some low- to mid-altitude areas such as the Morogoro region (eastern zone). Many CB landraces are still grown in Tanzania, despite low yield, because they perform predictably under the local conditions and meet peoples’ socio-economic criteria [4]. Since the 1950s, CB improvement (breeding) programs in Tanzania have been pursued to take advantage of the local landraces as well as CB germplasm maintained in international gene banks, such as CIAT (Colombia) and USDA Plant Germplasm System (USA) [4,38]. The breeding goals include resistance to viruses BCMV and BCMNV that cause significant yield losses [4]. Little information is available about other seedborne viruses in the CB crops in Tanzania.

Deep sequencing of virus-derived small interfering RNAs (vsiRNA) can be used to detect viruses [39]. This approach exploits the fundamental antiviral defense mechanism of plants, called RNA silencing or RNA interference (RNAi). It detects double-stranded RNA, such as the replicative forms of RNA viruses and secondary structures in single-stranded RNA viruses and the gene transcripts of DNA viruses [40–42]. The resulting 21, 22 and 24 nucleotide-long vsiRNAs are subjected to deep-sequencing, assembled into longer contiguous sequences (contigs) with bioinformatic tools and used as queries for searches in sequence databases [39]. Relative sequence identity of the contig(s) with previously described viral sequences identifies the virus present in the sample. The analysis can be continued by mapping the small-RNA reads from the sample to the homologous viral sequence(s) in the database, which confirms virus identification and provides an estimate of relatedness. Unknown viruses can be detected, too, as long as they show sufficient (>40%) sequence identity to regions of known viral genomes [39]. The method is time-efficient, affordable, generic, and detects all types of RNA and DNA viruses simultaneously in a single assay [39,43,44]. This detection method has been compared with next-generation sequencing of encapsidated and double-stranded long viral sequences in the recent study of Kutnjak et al. [45].

The aim of this study was to advance knowledge on the occurrence of seed-borne viruses transmitted via bean seeds in CB varieties currently grown in Nicaragua and Tanzania. In Nicaragua, the focus was on recently released CB varieties in La Compañia, because those varieties play or will play an important role in CB production in that country. In Africa, Tanzania is the second biggest CB producer in sub-Saharan Africa after Kenya [5], but surveys on seedborne viruses in CB crops have not been done recently. Therefore, CB seeds produced in three important bean growing regions were tested for viruses in Tanzania.

## Materials and methods

### Plant material

In Nicaragua, different CB varieties introduced to La Compañia for adaptability testing were surveyed and sampled in the field during the cropping season May-August, 2011. Plants displaying virus-like symptoms, if any, were marked. When mature, beans were harvested from four varieties (SEN46, SEN52, ‘CENTA Pipil’, MIB396). Three of these varieties had been bred in Zamorano, Honduras (SEN46, SEN52, ‘CENTA Pipil’) and one in CIAT, Colombia (MIB396). SEN46 and SEN52 have been released for cultivation under the names ‘INTA Caribe’ and ‘INTA Negro Precoz’, respectively, and MIB396 as ‘INTA Nutritivo’ [7]. One-hundred ten, 70, 60 and 10 bean seeds of ‘INTA Caribe’, ‘INTA Negro Precoz’, ‘INTA Nutritivo’ and CENTA Pipil, respectively, were shipped to University of Helsinki, Finland, under an import permit (no. 1605/0614/2010) obtained from the Finnish Food Safety Authority, Evira. Ten bean seeds per plant were sown in autoclaved soil in a greenhouse (temperature 18/27°C during day and night, respectively; photoperiod 12 h) in Helsinki. Prior to planting, the bean seeds were treated with fungicide (Topsin M, Berner, Finland) to eliminate possible seedborne fungi. Plants were grown for a month, leaves were sampled and samples were stored at –80°C.

Also, leaf samples were collected from some newly released CB varieties grown in La Compañia, including ‘INTA Cárdenas’, ‘INTA Rojo’, ‘INTA Seda 2’ (breeding line code NIC-704), ‘INTA Fuerta Sequia’ (RS-811-22), ‘INTA Frijol Norte' (628-SM-22-2) [7], and the breeding line XRAV-404. The collected leaf samples were dried on silica gel, airmailed in sealed paper bags to the University of Helsinki, Finland, under the aforementioned import permit, and tested for begomoviruses (genus *Begomovirus*; Geminiviridae) that are not seed-transmitted but can cause symptoms and yield losses in CB [46].

In Tanzania, bean seeds of a total of 38 CB landraces or improved varieties grown in three agro-ecological zones (Southern Highland, eastern and northern zones) were purchased from farmers in 15 administrative districts in 2015–2016. Landraces are varieties that were introduced to Tanzania at least 50 years ago. Their origin is not always known. Improved varieties have been developed by hybridization or selection processes to improve some of their traits. Bean seeds were planted in pots in insect-controlled screenhouses at Sokoine University of Agriculture or at Mikocheni Agricultural Research Institute. The soil used for planting bean seeds was heat-treated to kill any living organisms. The plants were sprayed with an insecticide (abamectin) regularly [47]. Screenhouses were visited daily for watering plants and confirming the absence of aphids and other insects. When close to flowering, leaf samples were taken from 30 CB plants per zone and immediately subjected to RNA isolation.

### RNA isolation

Total RNA was isolated from leaves of CB plants grown from 90 and 102 bean seeds harvested from Tanzania and Nicaragua, respectively, as described above. Leaves were ground with Geno/Grinder (SPEX SamplePrep, Metuchen, NJ, USA), or in liquid nitrogen in a mortar with a pestle, and 0.3 g leaf powder was quickly transferred into a 1.5-ml Eppendorf tube that was placed in liquid nitrogen. At University of Helsinki, total RNA was extracted from leaf samples using the Trizol protocol [48]. After precipitation, samples were centrifuged, supernatant was removed, and pellets were dissolved in 200 μl of nuclease-free water. At Mikocheni Agricultural Research Institute, total RNA was extracted using the CTAB method [49], and pellets were dissolved in 40 μl of nuclease-free water. RNA concentration and purity were determined with a Nanodrop 2000c UV–vis Spectrophotometer (Thermo Scientific, Wilmington, DE, USA). The quality of RNA in the samples was assessed visually by agarose gel electrophoresis with staining using ethidium bromide.

### Virus detection by siRNA sequencing and data analysis

An equal amount (1 μg) of total RNA from each leaf sample was combined to obtain six pools. As the samples from Nicaragua are concerned, the pools were based on the CB variety ‘INTA Caribe’ (36 samples) (GEN11), ‘INTA Negro Precoz’ (33 samples) (GEN12), and 33 RNA samples of ‘INTA Nutritivo’ (28 plants) and Centa Pipil (5 plants) (GEN13). RNA of the samples from the three agroecological zones in Tanzania were combined to form three pools of 30 samples each (HXH8-HXH10). From each RNA pool, 10 μg (Nicaragua) or 7 μg (Tanzania) of total RNA was sent to Fasteris SA (Plan-les-Outes, Switzerland) for deep sequencing. In Fasteris, samples were subjected to electrophoresis through an acrylamide gel, and the small RNAs of 1–43 (Nicaragua) or 1–44 nt (Tanzania) were purified from the gel. Single-stranded 3’ adapters and bar-coded 5’ adapters were ligated to the small-RNA oligonucleotides, reverse transcribed, and amplified by PCR to generate DNA colony template libraries. The libraries were purified and diluted to 10 nM concentration. Illumina Genome Analyzer was used for high-throughput DNA sequencing.

Initially, data were analyzed by GA pipeline (Broad Institute, USA) to convert images into sequences. Velvet software [50] was used to produce contigs by assembling the reads of 21–24 nt from high-throughput DNA sequencing data and sequences homologous to the contigs were searched in databases. Mapping of siRNA reads to the viral sequences identified by BLAST was carried out by MAQ (http://maq.sourceforge.net/index.shtml) or Bowtie (http://bowtie-bio.sourceforge.net/manual.shtml). However, since the VirusDetect pipeline became available, it was used to carry out the aforementioned analyses. VirusDetect is a new bioinformatics pipeline for efficient large-scale analysis of small-RNA datasets based on the aforementioned principles and algorithms [51]. VirusDetect is freely available at http://bioinfo.bti.cornell.edu/tool/VirusDetect/.

The small-RNA sequencing libraries and assembled contigs of viral sequences were deposited in European Nucleotide Archive (ENA) under the project (study accession) PRJEB19286 (S1 Table).

### Virus detection by RT-PCR

siRNA-based detection of viruses was confirmed by testing the total RNA in sample pools with reverse transcription–coupled PCR (RT-PCR). For RT, 1 μg of total RNA was treated with DNase (Promega, Madison, WI, USA). RT was carried out using *Moloney murine leukemia virus* reverse transcriptase (M-MLV RT) (Promega). DNase-treated RNA and 0.4 μg of random hexamer primers were mixed and heated at 70°C for 10 min. Each reaction was cooled immediately on ice, and 9 μl of a master mix was added. It contained 4 μl M-MLV RT 5× reaction buffer (Promega), 2 μl of 0.1 M DTT, 1 μl of 10 mM dNTP mix; 0.5 μl of 40 U/μl ribonuclease inhibitor (RNasin, Promega), and 1.5 μl of 200 U/μl M-MLV RT. The master mix was mixed well, and incubated at room temperature for 10 min and at 37°C for 1 h. Reactions were stopped by heating at 70°C for 10 min. Finally, 100 μl of nuclease-free water was added, and the samples were stored at –20°C.

PvEV-1 was detected with primers designed according to the conserved helicase-encoding region, as described by Segundo *et al*. [35]: forward primer PV3Up: 5’-GAATAATGGCATGTGAAGAC-3’, reverse primer PV4D: 5’-CAAAACCTGCTGGACCTA-3’; melting temperature 56°C; product size 374 bp. The consensus sequences recovered by MAQ and sequences from the NCBI database were aligned with MEGA5.1 [52] to design primers for amplification of the helicase-encoding region of PvEV-2 (forward primer P2-N2F2: 5’-GACTGTACTTGCTGTGGGCT-3’, reverse primer P2-N2R2: 5’-CGTCGGCAGAGAATTCCGTT-3’; melting temperature 60°C; product size 766 nt). Besides the RNA pools, single RNA samples (plants) included in the pools were tested by RT-PCR using the aforementioned primers. Phusion High-Fidelity DNA polymerase (Finnzymes, Espoo, Finland; or New England Biolabs Ltd., UK) was used according to the manufacturers’ instructions. PCR amplifications were carried out with forward and reverse primers (100 ng each) in GC buffer (Finnzymes) that improves the performance of Phusion DNA polymerase in amplification of GC-rich and long templates that might contain complex secondary structures. The PCR program for PvEV-1 was: 98°C for 1 min, followed by 30 cycles of 98°C for 10 s, 56°C for 30 s, and 72°C for 30 s, with a final step at 72°C for 5 min. The PCR program for PvEV-2 was similar to PvEV-1, except that an annealing temperature of 60°C was used. PCR products were analyzed by agarose gel electrophoresis (1% agarose; 140 V, 45 min), stained with ethidium bromide and visualized under UV light.

PCR products were purified using the E.Z.N.A. Gel Purification kit (Omega BioTech Inc., Norcross GA, USA) and sequenced at the Haartman Institute, University of Helsinki.

### DNA isolation for detection of begomoviruses by PCR

DNA was extracted from the dried leaf samples from Nicaragua using the quick method described by Wyatt & Brown [53]. Dried leaf tissue (0.05 g) was frozen in liquid nitrogen and ground in 2 ml of extraction buffer containing 50 mM Tris and 10 mM EDTA (pH 8). The extract was transferred to an Eppendorf tube and centrifuged in a tabletop centrifuge at full speed for 10 min. The supernatant (50 μl) was transferred to a new, sterile PCR tube and incubated on ice for 30 min. Subsequently, the extract was removed, the tube washed twice with 150 μl of 10 mM Tris and air dried for 40 min at room temperature. As a positive control, DNA was extracted from the leaves of *Abutilon pictum* ‘Thompsonii’ (Gillies) Walp. infected with *Abutilon mosaic virus* [54] and maintained at the Department of Agricultural Sciences, UH. The pair of degenerate primers PAL1v1978/PAR1c496 [55] designed for detection of geminiviruses was used to amplify a product of 1.1 kb covering the common region (CR) in DNA-A and DNA-B and parts of *AC1* (encodes the replication-associated protein) and *AV1* (encodes the coat protein), which corresponds to nucleotides 496–1978 in *Bean golden yellow mosaic Guatemala virus* (NCBI accession no. M91604) [56].

For direct PCR, DNA was not extracted but leaf sap was incubated in an Eppendorf tube, as described [53]. Fragments of viral DNA-A were amplified using two pairs of degenerate primers. Primers AV494 and AC1048 amplify a fragment of 576 bp that includes the coat protein gene [53]. The second pair of primers (PAL1v1978 and PAR1c496) amplified a region of begomovirus comprising the common region and the *AC1* and *AV1* open reading frames. The size of the amplified region varies from ca. 1.1 to 1.2 kb, depending on the begomovirus [55]. As a negative control, PCR was run in tubes not pre-incubated with a CB leaf extract. The PCR reaction mix (25 μl) contained 5 μl of 5× Phusion High-Fidelity reaction buffer, 0.5 μl dNTPs (10 mM), 1 μl MgCl_2_ (25 mM), 1 μl primers (10 μM), 0.5 U of Phusion High-Fidelity DNA polymerase, and MilliQ water to reach the final volume. DNA was amplified with a thermal cycler (Eppendorf Master Cycler Gradient). The program was: 1 min at 98°C, followed by 33 cycles of 10 s at 98°C, 30 s at 57 or 67°C (depending on the degenerate primers), and 30 s at 72°C, followed by 10 min at 72°C and cooling to 4°C. PCR products were analyzed by agarose gel electrophoresis (1.5%) using 1× TAE buffer [57]. Ethidium bromide was added to visualize DNA under UV light.

The PCR products of expected size were purified using the GeneJET Gel Extraction kit (Thermo Fisher) and sequenced at Macrogen Inc. (Seoul, Korea) or at the Institute for Molecular Medicine Finland (Helsinki, Finland), using the forward and reverse PCR primers.

## Results

### Viruses detected by small-RNA sequencing

Deep sequencing of the small RNAs in the different RNA pools from Nicaragua (1–43 nt) or Tanzania (1–44 nt) resulted in 6.1–13.3 million reads of 21–24 nt per pool. PvEV-1 and PvEV-2 were detected in all three sample pools from Nicaragua and two pools from Tanzania using the VirusDetect pipeline. PvEV-2, but not PvEV-1, was detected in one sample pool (HXH10) from Tanzania (Table 1).

**Table 1:** Viruses detected in the pools of common bean samples from Nicaragua (NI) and Tanzania (TZ) by VirusDetect.

Sample pool	Reference genome	Virus^a^	Coverage (%)^b^	Average depth^c^	Number of contigs	Size range of contigts (nt)
GEN11 (NI)	KT456287	PvEV-1	99.9	206.6	6	116–4526
	AB719398	PvEV-2	99.4	357.0	21	49–3008
GEN12 (NI)	KT456287	PvEV-1	99.9	352.6	4	78–12919
	AB719398	PvEV-2	98.5	252.4	28	50–2842
GEN13 (NI)	KT456287	PvEV-1	99.9	148.8	5	52–14061
	AB719398	PvEV-2	98.0	101.2	29	52–2842
HXH8 (TZ)	KT456287	PvEV-1	97.2	34.7	40	44–1000
	AB719398	PvEV-2	99.9	471.5	8	43–6277
HXH9 (TZ)	KT456287	PvEV-1	94.2	86.2	63	41–928
	AB719398	PvEV-2	87.9	119.6	99	39–722
	KJ534277	CPMMV	31.0	6.9	7	44–66
HXH10 (TZ)	KT456287	PvEV-1	ND	-	-	-
	AB719398	PvEV-2	88.9	14.1	77	38–978

^a^ PvEV-1 and PvEV-2, *Phaseolus vulgaris endornavirus 1* and *2*, respectively; CPMMV, *Cowpea mild mottle virus*.

^b^ Coverage of the full-length viral reference sequence.

^c^ The average number of times the nucleotides in the reference genome were covered by the small-RNA reads of the sample.

Mapping of the vsiRNA reads to the PvEV genomes showed high coverage and depth of coverage of the genomes (Table 1). Many contigs based on the reads of the three sample pools were nearly identical to the sequences of PvEV-1-Okada (AB719397) or PvEV-2-Okada (AB719398) characterized from the CB cultivar ‘Black Turtle Soup’ [25] and almost fully covered the genomes of PvEV-1 (13908 nt) and PvEV-2 (14820 nt). The longest single contig was 14061 nt (PvEV-1) (Table 1).

**Fig 1:**
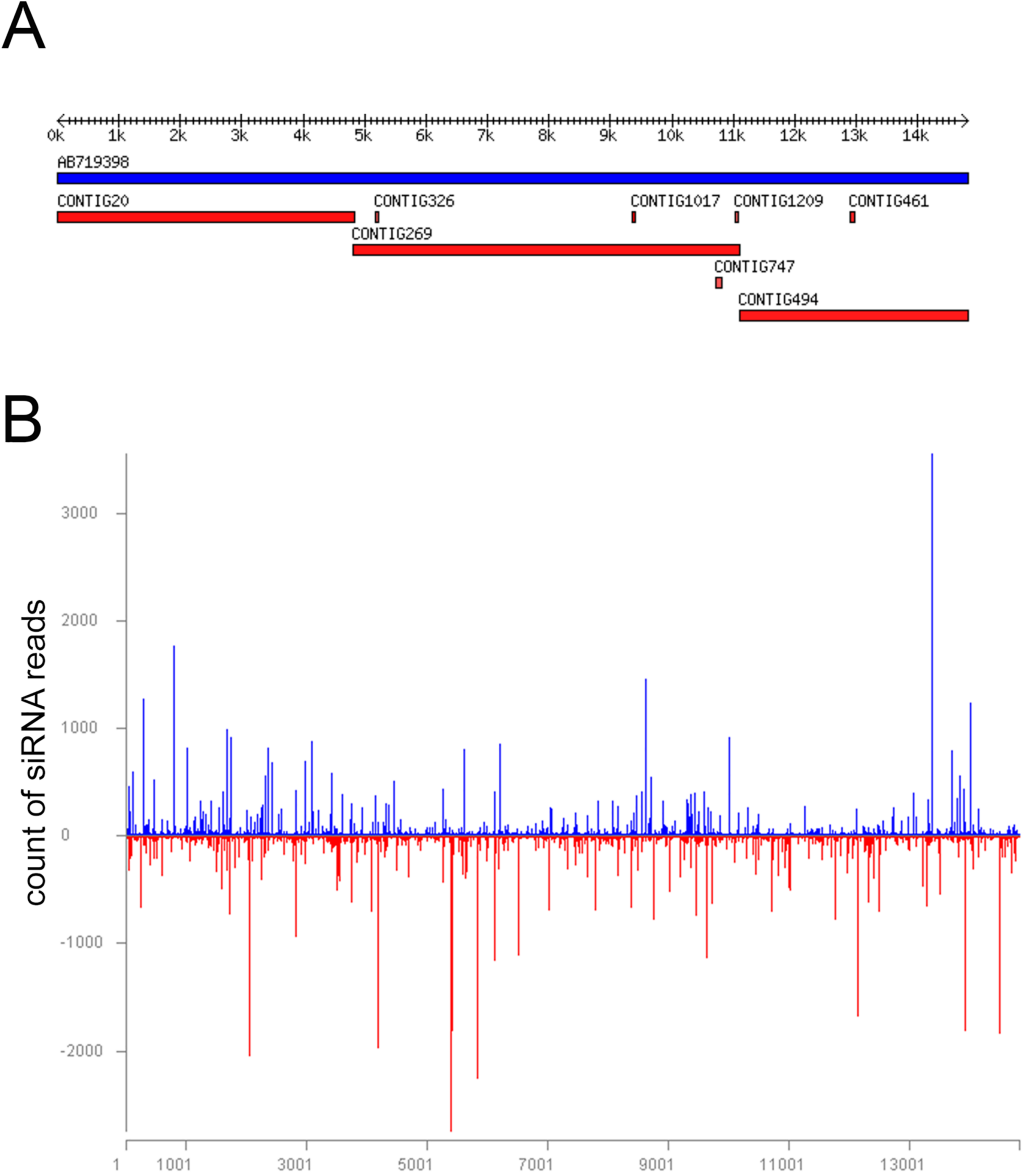
Identification of PvEV-2 in the sample pool HXH8 from the Southern Highland zone of Tanzania based on small-RNA deep sequencing. (a), Viral contigs (red bars) mapped to the sequence of PvEV-2-Okada (AB719398) [25] using VirusDetect [51]. Each nucleotide in the contigs was covered by siRNA reads at least 5 times. (b) The 21- to 24-nt reads mapped to the sequence of PvEV-2. The *x* axis and the scale below the figure depict the viral genome and nucleotide positions, respectively. The *y* axis indicates the number of siRNA reads derived from the coding strand (blue bars above the *x* axis) and complementary strand (red bars below the *x* axis).

The sample pool HXH8 from Tanzania contained 13 and 17 samples from improved varieties and landraces, respectively, collected in the Southern Highlands of Tanzania. Analysis by VirusDetect of the 21- to 24-nt reads identified PvEV-1 and PvEV-2. Sequences of contigs were nearly identical to PvEV-1-Melo or PvEV-2-Okada (Table 1). Forty contigs covered the genome of PvEV-1-Melo (97.5–100% identity), whereas three large contigs (3708–6277 nt) were sufficient to almost fully cover the genome of PvEV-2-Brazil (97–98% identity), as visualized using the MISIS program [58] in Fig 1.

Pool HXH9 contained 19 samples from improved CB varieties and 11 samples from landraces grown in the Arusha, Kilimanjaro or Manyara regions in northern Tanzania. PvEV-1 and PvEV-2 were detected, and the sequences of PvEV-1-Melo and PvEV-2-Okada were covered with 63 and 99 short contigs, respectively (S1 Table). Pool HXH10 included 12 samples from improved CB varieties and 18 samples from landraces grown in Morogoro in eastern Tanzania. PvEV-2 was readily detected and almost 90% of the genome of PvEV-2-Okada (AB719398) was covered with 77 contigs (97–100% identity) derived from the HXH10 pool. The largest and smallest contigs analyzed were 978 nt and 38 nt, with 98% and 100% identity, respectively, to PvEV-2-Okada. However, the contigs homologous to PvEV-1 were few and short.

The total of 17,863, 16,547 and 1,116 small-RNA reads obtained from the pools HXH8, HXH9 and HXH10 from Tanzania, respectively, aligned with the genome of PvEV-1-Melo. The coverage of PvEV-1-Melo was 97.2% (average depth 34.7) and 94.2% (average depth 86.2) for HXH8 and HXH9, respectively, whereas the number of vsiRNAs in HXH10 was too low for meaningful analysis. On the other hand, the numbers of small-RNA reads mapped to the genome of PvEV-2-Okada were 224,034 (HXH8), 18,844 (HXH9) and 6584 (HXH10). The coverage of the PvEV-2-Okada genome was 99.9% (average depth 472) in HXH8, 94% (average depth 86) in HXH9, and 89% (average depth 14) in HXH10. Only the first 2 nt (5’-end), last 6 nt (3’end) and 10 nt elsewhere in the genome could not be resolved (S1 Fig).

The similarity of the PvEV-1 and PvEV-2 sequences detected in Nicaragua and Tanzania with the previously determined PvEV-1-Okada and PvEV-2-Okada was confirmed by mapping the siRNA reads (21–24 nt) directly to the reference sequence. The numbers of reads mapped to PvEV-1-Okada and PvEV-2-Okada were 224,427 and 171,576, respectively, from pool GEN11 (‘INTA Caribe’), 131,834 and 244,122, respectively, from pool ‘INTA Negro Precoz’ (GEN12), and 95,201 and 71,208, respectively, from the pool of combined samples from ‘INTA Nutritivo’ (MIB396) and ‘Centa Pipil’ (GEN13). The coverage of PvEV-1 and PvEV-2 genomes with the small-RNA reads was 99.9% and 98.0–99.4%, respectively, depending on the sample pool. The average depth of coverage of the genomes of PvEV-1-Melo and PvEV-2-Okada was 149–353 and 101–357, respectively. Only the first 8 (5’-end) and last 8 (3’-end) nucleotides of PvEV-1-Nicaragua were not resolved. Similarly, the first 8 and last 11 nucleotides, and a few short regions (4–6 nt) elsewhere in the genome, could not be solved in PvEV-2-Nicaragua (S1 Fig).

No contig based on small-RNA data from samples of Nicaragua matched any sequence of pathogenic viruses. However, seven short contigs (44–66 nt) assembled from small-RNA reads of the pool HXH9 (Arusha, northern zone of Tanzania) were highly similar (95–98%) to CPMMV (NCBI accession no. KJ534277) and the vsiRNA reads of pool HXH9 covered 31% of the CPMMV genome. Studies on CPMMV are ongoing.

### Relatedness of the detected endornaviruses

**Fig 2:**
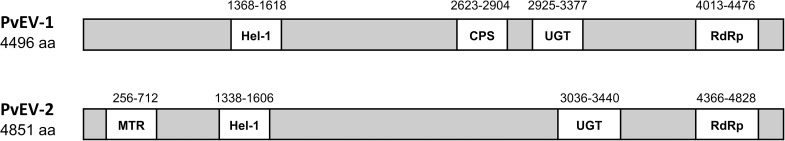
Conserved domains in the polyprotein encoded by PvEV-1 and PvEV-2 from Nicaragua. Numbers indicate the residues defining the conserved domains. Hel-1, helicase; CPS, putative capsular polysaccharide synthase; UGT, UDP-glycosyltransferase; RdRp, RNA-dependant RNA polymerase; and MTR, methyltransferase.

Endornaviruses differ in their genomic organization and conserved domains of the viral polyprotein [34]. The genomes of PvEV-1 and PvEV-2 encode a polyprotein of 4496 and 4851 amino acid residues, respectively, containing conserved domains such as the RNA helicase (Hel-1), UDP-glycosyltransferase (UGT) and RdRp domain. Furthermore, PvEV-2 contains a methyltransferase domain (MTR), whereas PvEV-1 contains a putative capsular polysaccharide synthase (CPS)-like domain [25]. The expected conserved domains were detected in the deduced polyprotein sequences of PvEV-1 and PvEV-2 detected in Nicaragua (Fig 2) and Tanzania.

The relatedness of PvEV-1 and PvEV-2 from Brazil, Nicaragua and Tanzania was compared using deduced amino acid sequences of the viral Hel-1 region assembled from small-RNA reads by VirusDetect. Hel-1 of PvEV-1-Okada was 98.9% identical to Hel-1 of PvEV-1 from Nicaragua and Tanzania. The Hel-1 sequences of PvEV-1 from Nicaragua was 99.2% identical to Hel-1 of PvEV-1 from Tanzania. On the other hand, Hel-1 of PvEV-2-Okada was 98.8% identical with PvEV-1 from Nicaragua and Tanzania. The Hel-1 of PvEV-2 from Nicaragua was 99.2% identical to Hel-1 of PvEV-2 from Tanzania.

### Detection of viruses by PCR

**Fig 3:**
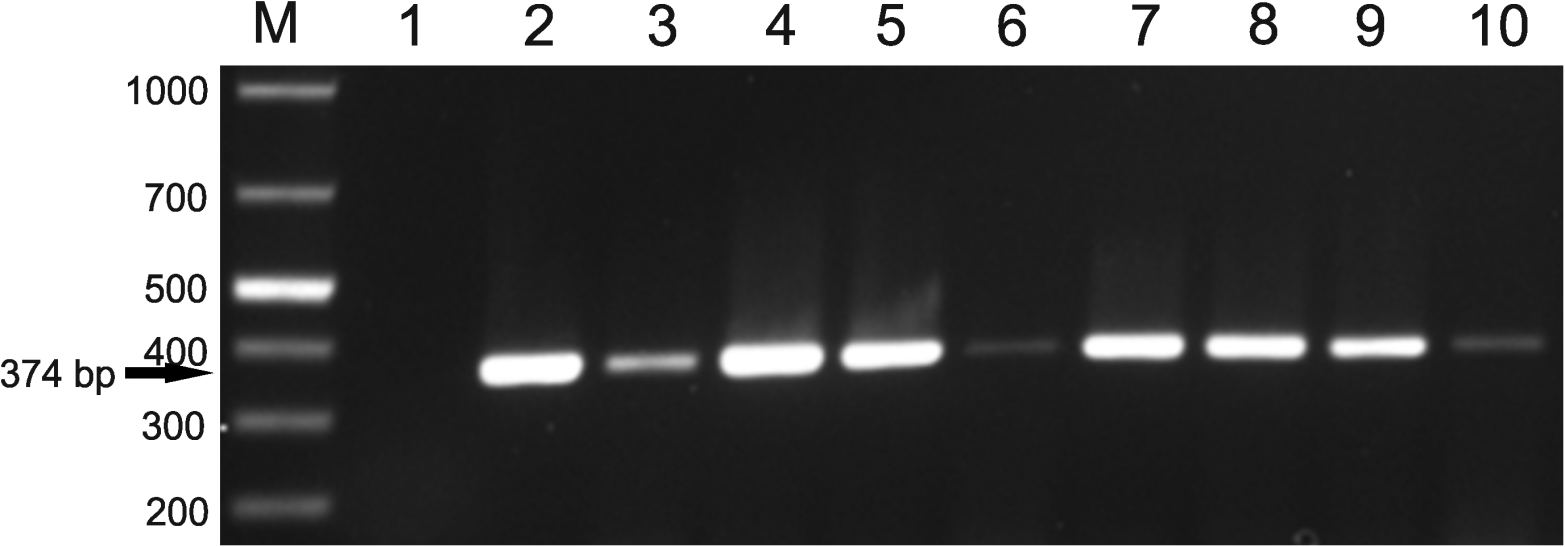
Detection of PvEV-1 by RT-PCR in common beans in Tanzania. In the list below, landraces are marked with asterisk (*). Other samples represent improved varieties (origin of samples shown in parenthesis). Lane labelled ‘M’ represents a O'GeneRuler 1 kb Plus DNA ladder. The expected size of PCR products was 374 bp. Lanes 1, ‘Njugu’* (Southern Highlands zone); 2, ‘pooled RNA’ (Southern Highlands zone); 3, ‘pooled RNA’ (Eastern zone); 4, ‘pooled RNA’ (Northern zone); 5, ‘Rosekoko’/’Lyamungu 85’ (Eastern zone); 6, ‘Salundi’ (Southern Highlands zone); 7, ‘E 36’ (Southern Highlands zone); 8, ‘Msafiri’* (Southern Highlands zone); 9, ‘Msafiri’* (Eastern zone); and 10, ‘Mshindi’ (Eastern zone).

Different improved CB varieties and landraces from the eastern and northern zones and Southern Highlands of Tanzania were tested for PvEV-1 by RT-PCR using primers designed to amplify the Hel-1-encoding sequence. PvEV-1 was detected in the randomly picked plants of CB landraces and improved varieties, as judged based on the expected size of PCR products assessed with agarose gel electrophoresis (Fig 3).

**Fig 4:**
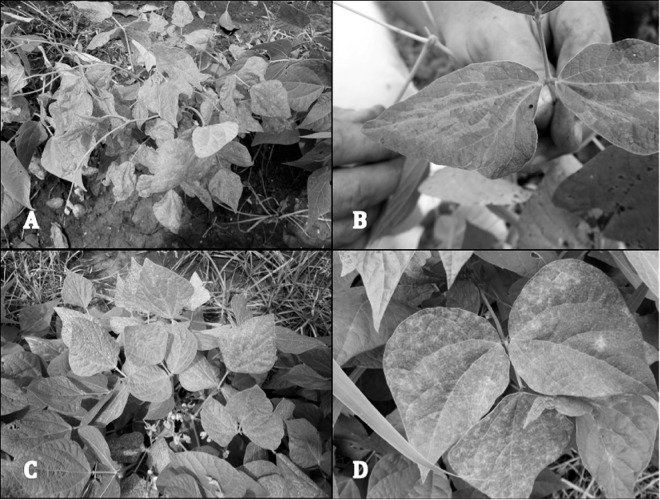
Symptoms observed in common bean plants in La Compañia, Nicaragua. (a), Stunting of the plant, malformation and blistering of leaves. (b), Mild epinasty and vein reversion. (c), Green-yellow chlorosis. (d), Green-yellow mosaic.

Concerning samples from Nicaragua, two plants each of the varieties ‘INTA Nutritivo’ and ‘Centa Pipil’ and four plants each of the varieties ‘INTA Caribe’ and ‘INTA Negro Precoz’ grown from bean seads in the greenhouse were tested for PvEV-1 and PvEV-2 by RT-PCR as above. The PCR products were of the expected size, as assessed with agarose gel electrophoresis. Comparison of the sequenced PCR products with consensus sequences built by MAQ and the sequences of PvEV-1-Okada and PvEV-2-Okada revealed that all tested plants of the four CB varieties from Nicaragua were positive for both endornaviruses. Furthermore, leaf samples were collected from six additional CB varieties showing virus-like symptoms in La Compañia (Fig 4) and tested by PCR using two pairs of universal primers designed for detection of begomoviruses [53,55]. All the tested 72 CB leaf samples were PCR negative. In contrast, both primer pairs amplified a product of the expected size in the positive control (*Abutilon pictum* infected with *Abutilon mosaic virus*). The authenticity of the PCR products was confirmed by sequencing.

### Symptoms and their association with viruses

Plants of CB varieties in Nicaragua and Tanzania showed many similar symptoms in the field (Fig 4). However, it was unlikely that the symptoms were associated with known pathogenic viruses transmitted in bean seeds, because the plants grown from seeds of such symptomatic plants produced virus-free plants–with exception of CPMMV in sample pool HXH9 from Arusha, Tanzania. Also the CB leaves sampled in Nicaragua and tested for begomoviruses using universal primers were virus-negative. The CB plants, which grew from bean seeds harvested in La Compañia, displayed no severe symptoms in the greenhouse in Helsinki. However, a few plants of the four varieties developed mild epinasty and vein reversion resembling the stronger symptoms observed in the fields of Nicaragua (Fig 4B and 4D) and Tanzania.

## Discussion

Beans were harvested from four new CB cultivars introduced to Nicaragua and a large number of CB landraces and improved varieties grown in three agricultural zones of Tanzania and used as seeds that were planted and grown under controlled conditions. Leaves of the plants were tested for seedborne viruses by small-RNA deep sequencing. In both countries, CB seeds were found to carry PvEV-1 and PvEV-2 that were vertically transmitted to the plants grown from the seeds. The small-RNA reads in the sample pools covered 14–471 fold the nucleotides in the previously characterized sequences of PvEV-1 and PvEV-2 [25], depending on the pool. Therefore, analysis of the new isolates by Sanger sequencing would have provided little value in terms of virus identification. A similar approach has been used to detect *Bell pepper endornavirus* in pepper (*Capsicum annuum* L.) [59]. However, other studies on endornaviruses, including PvEV-1 and PvEV-2 [25], have relied on isolation of viral double-stranded RNA from infected tissue, followed by RT to yield DNA, with subsequent analysis by next-generation sequencing ([28,60] and refs. therein). Comparison of our results with those of Okada *et al*. [25] suggests that both methods provide similar results. The modular arrangement of endornavirus genomes suggests that these viruses evolved via exchange of genomic domains [34]. According to our results and those of Okada *et al*. [25], the major difference in genome organization of PvEV-1 and PvEV-2 is the putative CPS domain found exclusively in PvEV-1 and the methyltransferase domain found only in PvEV-2.

A recent comprehensive survey for PvEV-1 and PvEV-2 in the Andes and Mesoamerica using RT-PCR for virus detection revealed that both viruses occur in CB cultivars and breeding lines in both domestication centers [36]. The single CB sample from Nicaragua included in that study was negative for PvEV-1 and PvEV-2. Their results showed that the incidence of both viruses is much higher in Mesoamerica than the Andes. Of 68 breeding lines/cultivars with Mesoamerican origin, 63 were co-infected with both viruses (the remaining 5 plants contained neither virus), whereas only 3 of 42 breeding lines/cultivars with Andean origin were infected with one or both viruses [36]. In our study, the four tested, modern CB cultivars grown in Nicaragua carried PvEV-1 and PvEV-2. ‘INTA Pipil’ has been bred in Samorano, Honduras, whereas the other cultivars have been bred in CIAT, Colombia. Some of these cultivars have common ancestors [7]. It is therefore challenging to categorize the four CB cultivars according to the domestication center. This is also the case with CBs in Tanzania, where large numbers of CB varieties are grown, but the origin of most of them is unknown [3,4,38]. Testing 30 CB plants grown from bean seeds harvested from each of the three surveyed zones important for CB production in Tanzania revealed that PvEV-2 exist in all the three zones, and PvEV-1 in at least two of them. Only few short contigs homologous to PvEV-1 were detected in the pool of samples from Morogoro, suggesting that PvEV-1 is not common there for reasons that could be interesting to elucidate. In Spain, an extensive survey of viruses in green beans (*P*. *vulgaris*) revealed that all 422 tested crops carried PvEV [35], more specifically PvEV-1 based on the studies of Okada *et al*. [25]. Vertical spread of endornaviruses via pollination [34] might facilitate spread of PvEV-1 and PvEV-2 among the CB varieties, elite breeding lines and new cultivars growing nearby. Hence, PvEVs may be more common in CB crops than currently known, which is pointed out by the findings made in Nicaragua and Tanzania in our present study.

The surveys of Segundo *et al*. [35] for a large number of viruses in green beans grown in greenhouses in Spain over several years detected no pathogenic virus in 61% of the plants displaying virus-like symptoms, regardless of whether serological or PCR-based methods were used. Examples of symptoms not associated with pathogenic viruses were illustrated and closely resembled some of the symptoms we observed in the CBs grown in La Compañia (Fig 4) and in Tanzania. Leaves of CB cultivars grown in the field and sampled in Nicaragua were tested for begomoviruses by PCR using universal primers, and results were negative, which excludes begomoviruses as the cause of the observed symptoms. Bacterial and fungal pathogens infecting roots and vascular tissues may cause chlorosis and growth disorders resembling symptoms of viruses, as illustrated by Hall [61]. Furthermore, symptoms may be associated with genetic disorders, problems with application of fertilizers, pesticides or herbicides and/or non-optimal growth conditions. For example, the mild physiological and phenotypic changes observed in the greenhouse in Finland might have been associated with lower temperature and light intensity than in the field in Nicaragua. On the other hand, it is also possible that some of the symptoms were associated with unknown viruses that are too distinct from the known viruses to be detected with the methods used in our study. Finally, symptoms may have been caused by a combination of several of the aforementioned factors.

The CB plants were grown from harvested, dry beans in vector-proof greenhouses or screenhouses in our study and only seedborne viruses, if any, were expected to occur in them. Besides PvEVs—and CPMMV in one sample pool in Tanzania—no other seedborne viruses were detected. Nevertheless, a few plants developed very mild epinasty and vein reversion on leaves, as also noticed in a few plants grown in the fields of Nicaragua and Tanzania. Contribution of PvEVs to the symptoms cannot be fully excluded because PvEV-free controls of the same CB varieties were not available for comparison. In general, the impact, if any, of endornaviruses on their hosts remains elusive in most cases [25,34]. Considering the efficient replication of PvEV genome in host cells, reflected by accumulation of high amounts of PvEV-derived small-RNAs, it seems plausible that PvEV-infected plants suffer a loss of energy and metabolites needed for its own physiology. Furthermore, endornaviruses are obviously not eliminated by the antiviral defense but seem to persist in plants over generations, which implies that endornaviruses interfere with or partially circumvent RNAi-based antiviral defense in the host–an intriguing issue for future study on endornaviruses. Interference with RNAi by viruses can interfere also with cellular RNAi-mediated gene regulation, which causes physiological disturbance and symptoms often displayed by virus-infected plants ([62]; and refs. therein) and, possibly, endornavirus-infected plants.

Indeed, there is evidence that endornaviruses can disturb physiology of the host plant. Cytoplasmic male sterility in faba bean (*Vicia faba* L.) is associated with *Vicia faba endornavirus* (VfEV). VfEV was originally referred to as a cytoplasmic male sterility–associated high-molecular-weight RNA. When Grill and Garger [63] transmitted VfEV to fertile faba beans using dodder (*Cuscuta subinclusa* Durand & Hilg.) as a bridge, the recipient plants maintained the VfEV RNA and became male-sterile. The virus-like features of the cytoplasmic male sterility–associated RNA became established later [64]. Because studies of VfEV have shown that endornaviruses can affect host physiology, it should be possible to observe changes in gene expression in the infected plants. Indeed, during preparation of this paper, Khankhum *et al*. [65] reported that expression of 84 genes is downregulated and 48 genes upregulated in the plants of CB cultivar ‘Black turtle soup’ co-infected with PvEV-1 and PvEV-2, as compared with ‘Black turtle soup’ free of the two endornaviruses. The genes affected were mainly associated with oxidation-reduction (redox) processes involved, e.g., in plant response to pathogen infection.

The rate of seed transmission of the pathogenic viruses BCMV, BCMNV, CMV, CPMMV and *Southern bean mosaic virus* can vary greatly depending on the CB cultivar, e.g., from 1% to 54% with BCMV [66]. Seed transmission depends on many physiological and developmental functions of the host plant. In general, transfer of virus from seed to seedling requires infection of the embryo, for which the physiological ‘window’ remains open only for a short time [9]. Furthermore, meristematic tissues and seed primordia mount a strong antiviral defense, which may eliminate the virus [67,68]. These bottlenecks could be tightened by plant breeding to further limit seedborne transmission. The resistance genes introgressed to the CB cultivars tested in the present study include i) the recessive gene *bgm-1* for resistance to *Bean golden yellow mosaic virus* (BGYMV; previously type II bean golden mosaic virus) [69–71], ii) a quantitative trait locus that controls resistance to BGYMV and can be selected using the sequence-characterized amplified region marker SW12 [72], and iii) the dominant gene *I* for hypersensitive resistance to BCMV, which can be selected using the sequence-characterized amplified region marker SW13 [73,74]. According to Ferrufino [7], cultivars ‘INTA Caribe’, ‘INTA Rojo’ and the breeding line X-RAV-404 carry all three resistance factors. Cultivars ‘INTA Frijol Norte’, ‘INTA Fuerte Sequía’, ‘INTA Pipil’ and ‘INTA Seda 2’ contain the resistance genes *I* and *bgm-1*. ‘INTA Cárdenas’ carries the gene *I* and the quantitative trait locus SW12. ‘INTA Negro Precoz’ contains *bgm-1*, whereas ‘INTA Nutritivo’ carries the gene *I* [7]. Progress in improving CB varieties in Tanzania by breeding for virus resistance includes combining the recessive resistance gene *bc-1*^*2*^ and the dominant gene *I* to protect CB against BCMV and BCMNV and circumvent the temperature-sensitive systemic vascular necrosis (black root) response caused by BCMNV in CB varieties varying the gene *I* [4,38]. The low incidence of seedborne infections of CB plants by pathogenic viruses in Nicaragua and Tanzania may be attributable to new, virus-resistant CB varieties released by breeding programs [75].

## Supporting information

S1 Table Accession numbers of the small-RNA sequencing libraries and assembled contigs of viral sequences deposited in European Nucleotide Archive (ENA) under the project (study accession) PRJEB19286. (DOC)

S1 Fig Alingment of the *Phaseolus vulgaris endornavirus 2* (PvEV-2) sequence (AB719398) determined by Sanger sequencing (Okada *et al*., 2013) with the sequences of PvEV-2 from common beans in Nicaragua (PvEV-2-SEN) and Tanzania (PvEV-2-Tan) reconstructed by assembly of virus-derived small-RNAs. The translation start and stop codons of the open reading frame are highlighted in green and yellow, respectively. The unresolved nucleotides of PvEV-2-Nicaragua and PvEV-2-Tanzania are marked with ‘*N*’.
(DOCX)
