# Correction: Pathogenic seedborne viruses are rare but *Phaseolus vulgaris* endornaviruses are common in bean varieties grown in Nicaragua and Tanzania

**DOI:** 10.1371/journal.pone.0184263

**Published:** 2017-08-29

**Authors:** Noora Nordenstedt, Delfia Marcenaro, Daudi Chilagane, Beatrice Mwaipopo, Minna-Liisa Rajamäki, Susan Nchimbi-Msolla, Paul J. R. Njau, Deusdedith R. Mbanzibwa, Jari P. T. Valkonen

Fig 4 incorrectly appears in black and white. Please see the corrected Fig 4 here.

**Fig 4:**
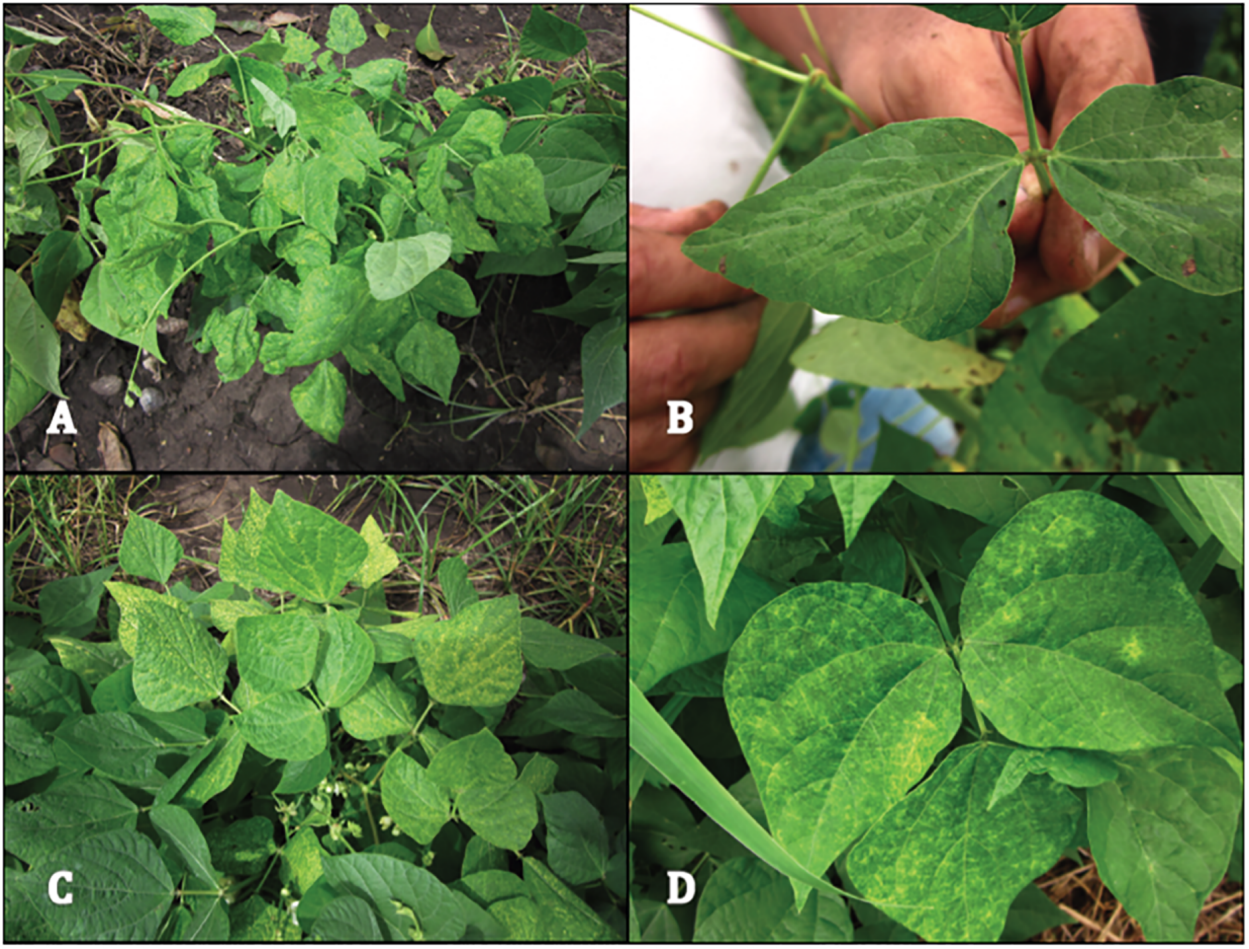
Symptoms observed in common bean plants in La Compañia, Nicaragua. (a), Stunting of the plant, malformation and blistering of leaves. (b), Mild epinasty and vein reversion. (c), Green-yellow chlorosis. (d), Green-yellow mosaic.
