# Supplementary material for: Pathogenic seedborne viruses are rare but Phaseolus vulgaris endornaviruses are common in bean varieties grown in Nicaragua and Tanzania
Source: PLoS One. 2017 May 25;12(5):e0178242. doi: 10.1371/journal.pone.0178242 (PMC5444779; doi:10.1371/journal.pone.0178242)
Supplement: S1 Table — (DOC) [file pone.0178242.s001.doc]

**SI Table**. Accession numbers of the small-RNA sequencing libraries and assembled contigs of viral sequences deposited in European Nucleotide Archive (ENA) under the project (study accession) PRJEB19286 (<http://www.ebi.ac.uk/ena>).

Study unique name: ena-STUDY-UNIVERSITY OF HELSINKI-02-02-2017-11:20:56:010-7

Study accession: PRJEB19286

**a) siRNA data**

**siRNA pool** **Sample accession Experiment accession Run accession**

HXH8 ERS1544419 ERX1880640 ERR1818138

HXH9 ERS1544420 ERX1880641 ERR1818139

HXH10 ERS1544421 ERX1880642 ERR1818140

GEN11 ERS1544422 ERX1880643 ERR1818141

GEN46 ERS1544423 ERX1880644 ERR1818142

GEN52 ERS1544424 ERX1880645 ERR1818143

**b) Assembled contigs**

**Sample acc. Analysis acc.**

PvEV-1-SEN46 ERS1544423 ERZ389521

PvEV-2-SEN46 ERS1544423 ERZ389522

PvEV-2-Tanzania ERS1544419 ERZ389523
