# Supplementary material for: Pathogenic seedborne viruses are rare but Phaseolus vulgaris endornaviruses are common in bean varieties grown in Nicaragua and Tanzania
Source: PLoS One. 2017 May 25;12(5):e0178242. doi: 10.1371/journal.pone.0178242 (PMC5444779; doi:10.1371/journal.pone.0178242)
Supplement: S1 Fig — The translation start and stop codons of the open reading frame are highlighted in green and yellow, respectively. The unresolved nucleotides of PvEV-2-Nicaragua and PvEV-2-Tanzania are marked with ‘N’. (DOCX) [file pone.0178242.s002.docx]

**S1 Fig.** Alingment of the *Phaseolus vulgaris endornavirus 2* (PvEV-2) sequence (AB719398) determined by Sanger sequencing (Okada *et al*., 2013) with the sequences of PvEV-2 from common beans in Nicaragua (PvEV-2-SEN) and Tanzania (PvEV-2-Tan) reconstructed by assembly of virus-derived small-RNAs. PvEV-2-TAN corresponds to sample pool HXH8, whereas PvEV-2-SEN corresponds to sample pool GEN11 (see Table 1). The translation start and stop codons of the open reading frame are highlighted in green and yellow, respectively. The unresolved nucleotides of PvEV-2-Nicaragua and PvEV-2-Tanzania are marked with ‘*N*’.

1 100

AB719398 GATA*ATAATT* *TGATTGTCGT* *CCAATGGCTA* *GAAAACTTGA* *CAAGGTCTCG* *TTTTTAAAGT* *CTTTGGGTGG* *TTCACACCAC* *CCGAAAATTC* *AAGCTTCTCA*

PvEV-2-Tan AG*......* *....*C*.....* *..........* *..........* *..........* *..........* *..........* *..........* *..........* *..........*

PvEV-2-SEN NNNNNNNN*..* *..........* *..........* *..........* *..........* *..........* *..........* *..........* *..........* *..........*

Consensus ....*ataa..* *....t.....* *..........* *..........* *..........* *..........* *..........* *..........* *..........* *..........*

101 200

AB719398 *TGAGCACACG* *AGCAATCGTG* *TGCTCATGAG* *AAAATTAAAT* *AAAAGAAATG* *AATGCTCTGC* *AAGAAACGTA* *TACAGACAGA* *GCA*G*TTATAA* *AGTAAAACTA*

PvEV-2-Tan *..........* *..........* *..........* *..........* *..........* *..........* *..........* *..........* *...A......* *.....*G*....*

PvEV-2-SEN *..........* *..........* *..........* *..........* *..........* *..........* *..........* *.....*G*....* *...A.*C*....* *..........*

Consensus *..........* *..........* *..........* *..........* *..........* *..........* *..........* *.....a....* *...a.t....* *.....a....*

201 300

AB719398 *CACTCAAGTA* *AGACCGTTGT* *ACCAGCGGTT* ***ATG****GCAGCCA* *GACAAGGGGA* *AGAATTGGAA* *TTCTTG*C*AAT* *TTGGTTATGA* *AGAATGTGTG* *CCGGACCTCA*

PvEV-2-Tan *..........* *..........* *.........*C *..........* *.......*A*..* *..........* *......G...* *..........* *..........* *..........*

PvEV-2-SEN *..........* *..........* *..........* *..........* *..........* G*.........* *......G...* *..........* *..........* *..........*

Consensus *..........* *..........* *.........t* *..........* *.......g..* *a.........* *......g...* *..........* *..........* *..........*

301 400

AB719398 *ACAAAATAGA* *CATACGCACA* *ATTCGGTCTA* *TGATGCAAC*A *ATTCGAACAA* *GGACATTGCC* *ACATAGATAT* *CACACCGGAG* *GAAGTTCAGG* *CAACGTTGCA*

PvEV-2-Tan *..........* *..........* *..........* *.........T* *..........* *..........* *..........* *..........* *..........* *..........*

PvEV-2-SEN *..........* *..........* *..........* *........*T*T* *..........* *..........* *..........* *..........* *..........* *.......*R*..*

Consensus *..........* *..........* *..........* *........ct* *..........* *..........* *..........* *..........* *..........* *.......g..*

401 500

AB719398 *CAATTGTCAA* *CTACAAAGCA* *CAGAGCCGTA* *TTACGGTGCC* *CCTGGAAAAC* *TGTATTACAA* *GCATTGTCGA* *CCTTTGTTGA* *CTCCAAGATC* *CAAAGAAGAT*

PvEV-2-Tan *..........* *..........* *..........* *..........* *..........* *..........* *..........* *..........* *..........* *..........*

PvEV-2-SEN *..........* *..........* *..........* *..........* *..........* *..........* *.........*G *..........* *..........* *..........*

Consensus *..........* *..........* *..........* *..........* *..........* *..........* *.........a* *..........* *..........* *..........*

501 600

AB719398 *GGAACAACAA* *CCGAAGCTGC* *CAGGGAC*C*TG* *TTACTGGGCA* *TAATTA*G*GAA* *ATTCGGCGGA* *TCCGTTTTAG* *GAATGCTGTA* *TCAGGACTGC* *GCCAACAATG*

PvEV-2-Tan *..........* *..........* *.......T..* *..........* *......A...* *..........* *..........* *..........* *......*T*...* *..........*

PvEV-2-SEN *..........* *..........* *.......T..* *..........* *......A...* *...*T*......* *..........* *..........* *..........* *..........*

Consensus *..........* *..........* *.......t..* *..........* *......a...* *...c......* *..........* *..........* *......c...* *..........*

601 700

AB719398 *CCAACATGAC* *GCCCCACGTT* *TACAGGCAGG* *G*G*AATCGTTA* *TGGATTTAAA* *GCCATGGAAA* *TGGCACAGCC* *CGACGCAGCA* *ACACTGTGTT* *GCTACTGCAA*

PvEV-2-Tan *..........* *..........* *..........* *.A....*T*...* *..........* *..........* *..........* *..........* *..........* *..........*

PvEV-2-SEN *..........* A*.........* *..........* *.A...*A*....* *...*G*..*C*...* *..........* *..........* *..........* *..........* *..........*

Consensus *..........* *g.........* *..........* *.a...cg...* *...a..t...* *..........* *..........* *..........* *..........* *..........*

701 800

AB719398 *AACTGTCAAT* *TTTGTTTGGT* *TGTATGGTGA* *GACCGACGGT* *CTTCAAAAAT* *CACGATGTGG* *GGTTTGCATG* *ACCCCACTGC* *TATTAGAACA* *CGCAGTGCCT*

PvEV-2-Tan *..........* *..........* *..........* *...*A*......* *..........* *..........* *..........* *..........* *..........* *..........*

PvEV-2-SEN *..........* *..........* *..........* A*.........* *..........* *..........* *..........* *..*T*.......* *........*T*.* *..........*

Consensus *..........* *..........* *..........* *g..c......* *..........* *..........* *..........* *..c.......* *........c.* *..........*

801 900

AB719398 *GAACACATAA* *TGTGGCCCAT* *GCTAACAGTT* *CACGACATTA* *CGCAATTGCA* *TGTAGGGGCA* *ACAGAGTTGC* *AAAATGCAGC* *AATCACCTTG* *CAACTAGACG*

PvEV-2-Tan *..........* *..........* A*...*G*.....* *..........* *..........* *..........* *..........* *..........* *..........* *..........*

PvEV-2-SEN *..........* *..........* *..........* *........*C*.* *..........* *...*T*......* *..........* *..........* *..........* *..........*

Consensus *..........* *..........* *g...a.....* *........t.* *..........* *...a......* *..........* *..........* *..........* *..........*

901 1000

AB719398 *GACAACCACC* *AATAACAACT* *AAAACAATGA* *GTAAGTTAAT* *AGAACAGGAT* *TTCAAAAACA* *TGAGGTCAAT* *CATGTCAAGG* *GC*A*ATGGCCA* *AAACTGTGTA*

PvEV-2-Tan *..........* *..........* *..........* *..........* *..........* *..........* *..........* *.........*A *..T.......* *..........*

PvEV-2-SEN *..........* *.........*C *..........* *.......*G*..* *..........* *..........* *..........* *..........* *..T.......* *..........*

Consensus *..........* *.........t* *..........* *.......a..* *..........* *..........* *..........* *.........g* *..t.......* *..........*

1001 1100

AB719398 *CGTCCCAAAT* *GGTATGACTG* *ATTTTCAGCA* *CAACCATCTG* *ACCGGCCAAG* *TGCCAGCCTA* *CAAGTTGATT* *AGAACCAATG* *CGATGCCCAA* C*CCGCATGCC*

PvEV-2-Tan *..........* *..........* *..........* *..........* *..........* *..........* *..........* *..........* *..........* *T.........*

PvEV-2-SEN *..........* *........*C*.* *.*C*........* *..........* *..........* *..........* *..........* *..........* *..........* *T..*R*......*

Consensus *..........* *........t.* *.t........* *..........* *..........* *..........* *..........* *..........* *..........* *t..g......*

1101 1200

AB719398 *ATGTTAGCAG* *CGGAGCGTAG* *AATCGCAACA* *ATCATGTTTG* *CAGATAAACG* *TGGGCCTGGA* *CAGACAATAT* *TGGACGTCGG* *ATCCAGGAAA* *AACTTGACTG*

PvEV-2-Tan *..........* *..........* *..........* *..........* *..........* *..........* *..........* *.......*T*..* *..........* *..........*

PvEV-2-SEN *..........* *..........* *..........* *..........* *..........* *...*A*......* *..........* *..........* *..........* *.....*A*....*

Consensus *..........* *..........* *..........* *..........* *..........* *...g......* *..........* *.......c..* *..........* *.....g....*

1201 1300

AB719398 *GGCTGAAGGA* *GTGGCACGGC* *ATGGGACCAA* *TAATGGACTG* *GCGTGACGTG* *GAACGGTACA* *ATGACAAACC* *ACTGAACACA* *AATATTTGCG* *ACCACACCGT*

PvEV-2-Tan *..*T*.......* *..........* *..........* *..........* *..........* *..........* *..........* *..........* *..........* *..........*

PvEV-2-SEN *..........* *..........* *.........*G *..........* *..........* *..........* *..........* *.....*G*....* *..........* *..........*

Consensus *..c.......* *..........* *.........a* *..........* *..........* *..........* *..........* *.....a....* *..........* *..........*

1301 1400

AB719398 *GAGAACTTGC* *GAATGTATGC* *AAGGGAAAAA* *GCCAATGGTG* *ATGTGCATAG* *ATTCCATTTA* *TGACATTGAT* *CCATGTGACG* *TACTGAAATT* *AATGCATACT*

PvEV-2-Tan *..........* *..........* *..........* *..........* *..........* *..........* *..........* *..........* *..........* *..........*

PvEV-2-SEN *..........* *..........* *..........* *..........* *..........* *..........* *..........* *..........* *....*A*.....* *..........*

Consensus *..........* *..........* *..........* *..........* *..........* *..........* *..........* *..........* *....g.....* *..........*

1401 1500

AB719398 *GTCCAGACCA* *ATCAACTGTT* *CTTCTGTGTG* *AGTACAGCAG* *AAGTCGATTT* *TGAATCCAC*C *AGCGGACTAT* *TGGCACACGG* *GCAAGGAATG* *TGGTCTAGAC*

PvEV-2-Tan *..........* *..........* *..........* *..........* *..........* *.........T* *.....*G*....* *..........* *..........* *..........*

PvEV-2-SEN *..........* *..........* *..........* *..........* *..........* C*........T* *..*Y*.......* *..........* *..........* *..........*

Consensus *..........* *..........* *..........* *..........* *..........* *t........t* *..c..a....* *..........* *..........* *..........*

1501 1600

AB719398 *ACAAAGATAA* *GCTAGTCACA* *AGTCTACGAG* *GCGATGACAG* *ACCTTATGTC* *AATAGCTGGA* *AATTGACCAA* *ACTGTGGTCG* *ACCGCCGATC* *TCATTCAAAT*

PvEV-2-Tan *..........* *..........* *..........* *..........* *..........* *..........* *..........* *..........* *..........* *..........*

PvEV-2-SEN *..........* *..........* *..........* *..........* *..........* *..........* *..........* *..........* *..........* *..........*

Consensus *..........* *..........* *..........* *..........* *..........* *..........* *..........* *..........* *..........* *..........*

1601 1700

AB719398 *TAACCAAGTA* *GAACTAAATC* *TGCAGACCAT* *TAAAGTGCTC* *GGGAACCATC* *TTTTGAGGGT* *GCTAACCATA* *GTCCCGAGCG* *GAATGGACTG* *TTTACGAACT*

PvEV-2-Tan *..........* *..........* *..........* *..........* *..........* *..........* *..........* *..........* *..........* *..........*

PvEV-2-SEN *..........* *..........* *..........* *..........* *.....*T*....* *..........* *..........* *..........* *..........* *..........*

Consensus *..........* *..........* *..........* *..........* *.....c....* *..........* *..........* *..........* *..........* *..........*

1701 1800

AB719398 *CGGCAATT*A*G* *ATCACAGGAC* *GATGACACAA* *GTGGACTTGG* *TGGCCCCAGT* *GATTGATGTT* *GA*G*AGTTGGC* *TCAATGTGTT* *TGGGGTACCA* *AAAATAAGCT*

PvEV-2-Tan *........G.* *..........* *..........* *..........* *..........* *.........*C *..A.......* *..........* *..........* *..........*

PvEV-2-SEN *........G.* *..........* *..........* *..........* *..........* *...*A*......* *..A.......* *..........* *..........* *..........*

Consensus *........g.* *..........* *..........* *..........* *..........* *...t.....t* *..a.......* *..........* *..........* *..........*

1801 1900

AB719398 *ACAAGCTAAC* *CAAATTGGAT* *TTGCAACTGT* *ACCGCGCATT* *GTATTCCCGC* *AATCTGACAG* *GGAGTCTGTC* *CTTCAATAAA* *TTGATCGA*G*T* *TTGGACTTGG*

PvEV-2-Tan *..........* *..........* *..........* *..........* *..........* *..........* *..........* *..........* C*.......A.* *..........*

PvEV-2-SEN *....*A*.....* *....*C*.....* *..........* *..........* *..........* *..........* *.....*T*....* *..........* *..*R*.....A.* *..........*

Consensus *....g.....* *....t.....* *..........* *..........* *..........* *..........* *.....c....* *..........* *t.g.....a.* *..........*

1901 2000

AB719398 *TTACGCTCAT* *GTAAAGTACA* *CCACAAGAAC* *ATCCACCATA* *ACACATCACC* *ACATTAGTGC* *GGATGACATC* *AGGTTACATG* *CAATGCTTGC* *ATGTGTGGCA*

PvEV-2-Tan *..........* *..........* *..........* *..........* *..........* *..........* *..........* *..........* *..........* *..........*

PvEV-2-SEN *..........* *..*G*.......* *..........* *..........* *..........* *..........* *..........* *..........* *..........* *..........*

Consensus *..........* *..a.......* *..........* *..........* *..........* *..........* *..........* *..........* *..........* *..........*

2001 2100

AB719398 *ACGAGACGCA* *AATTTGCTTG* *GGTCAAGGCA* *GTCACAGCGT* *TAAACACCAC* *ATCAAACGCG* *TTTGGCTT*A*G* *ACCCGATAGA* *AGT*G*GTCAAG* *CTCATGTCTA*

PvEV-2-Tan *..........* *..........* *..........* *..........* *..........* *..........* *........G.* *..........* *...A......* *..........*

PvEV-2-SEN *..........* *..........* *..........* *..........* *..........* *..........* *........G.* *..........* *...A......* *..........*

Consensus *..........* *..........* *..........* *..........* *..........* *..........* *........g.* *..........* *...a......* *..........*

2101 2200

AB719398 *TTGATCTAGC* *AGGGAAGTTG* *GTGAACGAAA* *TTTGGGGAGC* *CTTGTTGCCC* *GATGG*C*GCAC* *TCAAATCCAA* *ATTGAGTGGC* *ATGGTGGATA* *CTGTAAAAGC*

PvEV-2-Tan *..........* *..........* *..........* *..........* *..........* *.....T....* *..........* *..........* *..........* *..........*

PvEV-2-SEN *..........* *...*A*......* *.....*T*....* *..........* *..........* *.....T....* *.......*A*..* *.........*T *........*C*.* *.*A*........*

Consensus *..........* *...g......* *.....c....* *..........* *..........* *.....t....* *.......c..* *.........c* *........t.* *.t........*

2201 2300

AB719398 *ATGGATCAAT* *GACCCCTTCT* *GGGATGCACT* *CGAGCAGTTG* *AACGAAGGAT* *CAAAACGCTT* *TGAACCCAAT* *GTAGTGGTCT* *GGCCCATGTT* *AACTGCCGAG*

PvEV-2-Tan *..........* *..........* *..........* *..........* *..........* *..........* *..........* *.....*A*....* *..........* *..........*

PvEV-2-SEN *..........* *..........* *......*T*...* *..........* *..........* *..........* *..........* *..........* *..........* *..........*

Consensus *..........* *..........* *......c...* *..........* *..........* *..........* *..........* *.....g....* *..........* *..........*

2301 2400

AB719398 *ACCAGCGTAG* *AATCCGACTA* *TGTGTGTTGT* *CACCACAACC* *TGTGGTGTGA* *ACACGAGGCC* *AAACTGGAAG* *AGCTGTGCGA* *ATGCTGCAGA* T*TGGCCACAA*

PvEV-2-Tan *..........* *..........* *..........* *..........* *..........* *..........* *..........* *..........* *..........* *C.........*

PvEV-2-SEN *..........* *....*T*.....* *..........* *.........*A *..........* *..........* *..........* *..........* *..........* *C.........*

Consensus *..........* *....c.....* *..........* *.........c* *..........* *..........* *..........* *..........* *..........* *c.........*

2401 2500

AB719398 *AATTGAGTGA* *TAGTCAGTAT* *TGTCTGTGCT* *GCCGACCTCA* *GGCACCCAAA* *CACAAATGCG* *CGCACCACTG* *CTATGGAAAT* *CATGGAGATG* *TGAAACAGAA*

PvEV-2-Tan *..........* *..........* *..........* *..*A*.......* *..........* *..........* *..........* *..........* *..........* *..........*

PvEV-2-SEN *..........* *..........* *..........* *..........* *..........* *..*T*.......* *..........* *..........* *..........* Y*.........*

Consensus *..........* *..........* *..........* *..c.......* *..........* *..c.......* *..........* *..........* *..........* *t.........*

2501 2600

AB719398 *GGGCGAGACA* *GACTGTACTT* *GCTGTGGGCT* *ACGCACAGTA* *AATGGAGTGT* *GCAAGTGTTG* *TGTGAACAAA* *TTAGGCAAAT* *GGCCC*A*CACA* *GCAG*C*AAGGT*

PvEV-2-Tan *..........* *..........* *..........* *..........* *..........* *..........* *..........* *..........* *.....G....* *....G.....*

PvEV-2-SEN *........*Y*.* *..........* *..........* *..........* *..........* *..........* *..........* C*.........* *.....G....* *....G.....*

Consensus *........c.* *..........* *..........* *..........* *..........* *..........* *..........* *t.........* *.....g....* *....g.....*

2601 2700

AB719398 *GGCATCCCAC* *TCAAGTACCG* *CCGAGAGGAA* *ATCGACA*G*AG* *CAAAAAAAAT* *TGTCAAGTCG* *CGCACTACCA* *CACAAGAGTT* *ACCTCAAACA* *GCCCAAAAAA*

PvEV-2-Tan *..........* *..........* *..........* *.......A..* *..........* *...*T*......* *..........* *..........* *..........* A*.........*

PvEV-2-SEN *..........* *..........* *..........* *.......A..* *..........* *..........* *..........* *........*CC *..........* *......*G*...*

Consensus *..........* *..........* *..........* *.......a..* *..........* *...c......* *..........* *........tt* *..........* *g.....a...*

2701 2800

AB719398 *TGCCACCAGC* *TGGGACAGCA* *GG*C*GAAAGTG* *GCCCAACACC* *GCACGCTGAC* *AAACCAAGCG* *ATGAGCAAAA* *CCATTCTGGA* *AAAACAACGG* *AAGAAAGTCA*

PvEV-2-Tan *..........* *..........* *..*T*.......* *..........* *..........* G*.........* *..........* A*........*G *..........* *..........*

PvEV-2-SEN C*.........* *...*NNNNNNN NNNNNNN*...* *..........* *.......*A*..* *..........* *..........* *..*G*.....*A*.* *..........* *...*C*......*

Consensus *t.........* *...gacagca* *gg*.*gaaa...* *..........* *.......g..* *a.........* *..........* *c.a.....ga* *..........* *...a......*

2801 2900

AB719398 *AGCAGAACTC* *ACGATACTCG* *ACAAATGGAA* *ACAAGGTCAG* *GCCAGTTCAT* *CAGG*T*ACAAA* *AACAGACGTG* *GCCCAAGGCA* *TATCAA*A*CCT* *GAACCAACAA*

PvEV-2-Tan *..........* *..........* *..........* *..........* *..........* *....C.....* *..........* *..........* *......G*T*..* *..*G*.......*

PvEV-2-SEN *......*G*...* *..........* *..........* *.........*A *..........* *....C.....* *..........* *..........* *......G..*C *......*C*...*

Consensus *......a...* *..........* *..........* *.........g* *..........* *....c.....* *..........* *..........* *......gc.t* *..a...a...*

2901 3000

AB719398 *CCGAAGAAAA* *GTGATGGGCA* *AATGGATCAA* *CACGCCATGT* *CTACTGAAGA* *TGAGGTCGTG* *TTCAATGAAC* *TAAAAACATT* *AACCACCTTG* *GATTATGCTT*

PvEV-2-Tan *..........* *..........* *..........* *..........* *..........* *..........* *..........* *..........* *..........* *..........*

PvEV-2-SEN *.......*G*..* *..........* *..........* *..........* *..........* C*.........* *..........* *.*G*........* *...*T*......* *..........*

Consensus *.......a..* *..........* *..........* *..........* *..........* *t.........* *..........* *.a........* *...c......* *..........*

3001 3100

AB719398 *GTAATGTGAG* *GTACGGGTAC* *ACATACACAG* *TGACTGAGCA* *CTTGGGTGTG* *CCAAATGA*A*T* *GCAACCCGTT* *GAACCTGATC* *GGAATTAGTG* *AACTCAAATA*

PvEV-2-Tan *..........* *......*A*...* *..........* *..........* *..........* *........G.* *..........* *..........* *..........* *..........*

PvEV-2-SEN *..........* *..........* *..........* *..........* *..........* *........G.* *.......*A*..* *..........* *..........* *..........*

Consensus *..........* *......g...* *..........* *..........* *..........* *........g.* *.......g..* *..........* *..........* *..........*

3101 3200

AB719398 *TTTGCCGATC* *GG*A*AAAAAAT* *TGCACCCTCC* *CTCATGGTTA* *ACTGTAGTGG* *ACAGGGCGAG* *TGTGCCAGGG* *GATGGCATAT* *GTGGCTTCCA* *TGCAATCAAT*

PvEV-2-Tan *..........* *..*G*.......* *..........* *..........* *..........* *..........* *..........* *..........* *..........* *..........*

PvEV-2-SEN *..........* *..*R*.......* *..........* *..........* *......*A*...* *..........* *..........* *..........* *..........* *..........*

Consensus *..........* *..*.*.......* *..........* *..........* *......g...* *..........* *..........* *..........* *..........* *..........*

3201 3300

AB719398 *AGAGTGCTAG* *GGGGAAATTT* *ATCACTTGTG* *GAGATGCAGA* *GGGTGACCAG* *GCGGCAAACG* *GAATTCTCTG* *CCGACGAATT* *GTTGGACTAT* *TTGGCTTATT*

PvEV-2-Tan *..........* *..........* *..........* *..........* *..........* *..........* *..........* *..........* *..........* *..........*

PvEV-2-SEN *......*T*...* *..........* *.........*A *..........* *....*A*.....* *..........* *..........* *.......*G*..* A*.........* *..........*

Consensus *......c...* *..........* *.........g* *..........* *....g.....* *..........* *..........* *.......a..* *g.........* *..........*

3301 3400

AB719398 *TGGGGAAAAA* *TGGGATTATT* *CTGACAAAAG* *ACACAATAGC* *CAGTAACAAG* *ATTGATCCTT* *TTTCAGATCA* *ATATGTGTGT* *GTGCGCCACT* *GTGAAACCGA*

PvEV-2-Tan *..........* *..........* *..........* *..........* *..........* *..........* *..........* *..........* *..........* *..........*

PvEV-2-SEN *..........* *..........* *..........* *..........* *..........* *..........* *..........* *..........* *..........* *..........*

Consensus *..........* *..........* *..........* *..........* *..........* *..........* *..........* *..........* *..........* *..........*

3401 3500

AB719398 *TCCGAATCCC* *AAAAACCACT* *GGGAGCCGTG* *CACCGTCATA* *CAGACGGAAG* *ACATGGAGTA* *CACACCATGC* *TTTAACGGTT* *TGTTCAAACA* *GTCCCAAGTC*

PvEV-2-Tan *..........* *..........* *..........* *..........* *..........* *..........* *..........* *..........* *..........* *..........*

PvEV-2-SEN *..........* *..........* *..........* Y*.........* *..........* *..........* *..........* *.........*C *..........* *..........*

Consensus *..........* *..........* *..........* *c.........* *..........* *..........* *..........* *.........t* *..........* *..........*

3501 3600

AB719398 *AACACAAAAC* *AGTTGTGGAT* *GACAACAGCG* *ACAACGGAAA* *TTGAGCACAA* *AAACCAGGTG* *ATGCGAATTG* *TGCTGGAACT* *GATTAAAAGA* *GCCCAGCCCA*

PvEV-2-Tan *..........* *..........* *..........* *..........* *..........* *..........* *..........* *..........* *..........* *..........*

PvEV-2-SEN *..........* *..........* *......*G*...* *..........* *..........* *..........* *..........* *..........* *..........* *..........*

Consensus *..........* *..........* *......a...* *..........* *..........* *..........* *..........* *..........* *..........* *..........*

3601 3700

AB719398 *AACAGAGTGC* *AGCAGCTGCC* *AGTGAATTAA* *ACCTCAAGTT* *AATTGACACT* *CTACATGACA* *CATGGATGTC* *CAACAATGCA* *ACAATGGTAA* *ATGATCCTGC*

PvEV-2-Tan *..........* *..........* *..........* *..........* *..........* *..........* *..........* *..........* *..........* *.......*G*..*

PvEV-2-SEN *..........* *.........*Y *..........* *..........* *.........*C *..........* *..........* *..........* *..........* *..........*

Consensus *..........* *.........c* *..........* *..........* *.........t* *..........* *..........* *..........* *..........* *.......t..*

3701 3800

AB719398 *AGCAGGGTTG* *TTCCATTATA* *AAATACGAGC* *TGAAGACTAC* *GAGTACCTGG* *CATGCCTCAT* *AAACAGGAAA* *ATGACTGCGG* *ACATCAAGGA* *CGACTTAATT*

PvEV-2-Tan *..........* *..........* *..........* *..........* *..........* *..........* *..........* *........*A*.* *..........* *..........*

PvEV-2-SEN *..........* *..........* *..........* *..........* *.........*A *..........* *......*A*..*G *..........* *..........* *..........*

Consensus *..........* *..........* *..........* *..........* *.........g* *..........* *......g..a* *........g.* *..........* *..........*

3801 3900

AB719398 *GACCCACTGA* *ACATAGGGTT* *AATGCACGAC* *ACGGATATTG* *ACCAGTTGCG* *AAG*T*GC*A*TTA* *TTGCGAAATG* *TGGTGTACCA* *GTGCTGTCTC* *ATGTTGAATG*

PvEV-2-Tan *..........* *..........* *..........* *..........* *..........* *...C..G...* *..........* *..........* *......*C*...* *..........*

PvEV-2-SEN *..........* *..........* *.......*A*..* *.....*Y*...*A *..........* *...C..G...* *..........* *..........* *..........* *..........*

Consensus *..........* *..........* *.......g..* *.....t...g* *..........* *...c..g...* *..........* *..........* *......t...* *..........*

3901 4000

AB719398 *AAGGTGACGG* *TACAGAGCGC* *ATGACTGCAG* *AACTGTGGAC* *TAGCATGTCA* *ATTGACACCA* *CCAAAGTGGC* *GGGGGGACTG* *CGTATGCTAC* *AGTTGGGGGA*

PvEV-2-Tan *..........* *..........* *..........* *..*T*.......* *..........* *..........* *..........* *..........* *..........* *..........*

PvEV-2-SEN *..........* *...*C*......* *..........* *..........* *..........* *..........* *.*T*........* *..........* *..........* *.......*A*..*

Consensus *..........* *...a......* *..........* *..c.......* *..........* *..........* *.c........* *..........* *..........* *.......g..*

4001 4100

AB719398 *CACCAAGGTG* *AAAACTGGTG* *ATGTAATATC* *TGTAAACACG* *ATAAGTGGAC* *CTCAACACAG* *GCTAGTCTGG* *AAACACAACG* *GAAGATATTT* *CTGCAAAGAA*

PvEV-2-Tan *..........* *..........* *.*C*........* *..........* *..........* *..........* *..........* *..........* *..........* *..........*

PvEV-2-SEN *..........* *..........* *..........* *..........* *..........* *..........* *..........* *..........* *..........* *..........*

Consensus *..........* *..........* *.t........* *..........* *..........* *..........* *..........* *..........* *..........* *..........*

4101 4200

AB719398 *TGGATGCCCC* *CAAACCACGA* *AAAGTTGAGA* *ACAACATTGC* *ACATATGCAG* *GCAAAGCTTT* *AAGAGTGCCA* *TGATCCAAAT* *GTACTGCCTA* *TCACGAAATC*

PvEV-2-Tan *..........* *..........* *..........* *..........* *.......*T*..* *..........* *..........* *..........* *..........* *..........*

PvEV-2-SEN *..........* *..........* *..........* *..........* *..........* *..........* *..........* *..........* *..........* *..........*

Consensus *..........* *..........* *..........* *..........* *.......c..* *..........* *..........* *..........* *..........* *..........*

4201 4300

AB719398 *ACATTGAGTT* *TGACAAATTC* *AAACAATTAG* *TGGAGATGGC* *AGTGTGCACA* *TTGGGACCAG* *CAGGGAGTGG* *CAAAACAACA* *AAAATAGCAA* *GAGATTGGAC*

PvEV-2-Tan *..........* *..........* *..........* *....*C*.....* *..........* *..........* *..........* *..........* *..........* *..........*

PvEV-2-SEN *..........* *..........* *..........* *..........* *..........* *..........* *.*G*........* *..........* *..........* *..........*

Consensus *..........* *..........* *..........* *....g.....* *..........* *..........* *.a........* *..........* *..........* *..........*

4301 4400

AB719398 *CACCGATGAT* *TTGGCAATTG* *CGAGAACGAC* *AGTGGCGGTA* *ACATCACTGA* *GGGAAAAATT* *GAACTCACCA* *AAACAATTGG* *TGATGAGTCA* *CGAAAAATAC*

PvEV-2-Tan *..........* *..........* *..........* *..........* *..........* *..........* *..........* *..........* *..........* *..........*

PvEV-2-SEN *...*A*......* *..........* *.......*A*..* *..........* *..*G*.......* *..........* *.........*G *..........* *..........* *..........*

Consensus *...c......* *..........* *.......g..* *..........* *..a.......* *..........* *.........a* *..........* *..........* *..........*

4401 4500

AB719398 *AGTTTCACTC* *AACCACCAAC* *ACACAGGTTA* *GTAATAGATG* *AGTGCACCAT* *GTTTCCATGG* *TATGAGTTAT* *ATTTTTCTTT* *AACCACGCTG* *CCTACATCTT*

PvEV-2-Tan *..........* *.........*T *..........* *..........* *..........* *..........* *..........* *..........* *...*T*......* *..........*

PvEV-2-SEN *..........* *..........* *......*A*...* *..*G*.......* *..........* *...*C*......* *..........* *..........* *..........* *........*Y*.*

Consensus *..........* *.........c* *......g...* *..a.......* *..........* *...t......* *..........* *..........* *...c......* *........t.*

4501 4600

AB719398 *TGGTAATGTA* *CGGTGATCCA* *AACCAAATAA* *GTACAATAGA* *CACATACATG* *CTGGGTGGGG* *AGCGGATACT* *GGACAACATT* *GCAGATTACG* *TGCCAAACAA*

PvEV-2-Tan *..........* *..........* *..*T*.......* *..........* *..........* *..........* *..........* *..........* *..........* *..........*

PvEV-2-SEN *..........* *..........* *..........* *..........* *...*G*......* *..........* *..........* *..........* *..........* *.*A*........*

Consensus *..........* *..........* *..c.......* *..........* *...a......* *..........* *..........* *..........* *..........* *.g........*

4601 4700

AB719398 *GACACTGCTC* *AAGTCCACGT* *ATAGGTACGG* *GCCCAAGTTG* *TGTGGAATAC* *TCAATTCGAT* *CGTAGGTGAG* *ATAGTTAGCA* *ATGCTCCACA* *TGACACAATG*

PvEV-2-Tan A*.........* *..........* *..........* *..........* *..........* *..........* *......*A*...* *..........* *..........* *..........*

PvEV-2-SEN *..........* *..*A*.......* *..........* *..........* *..........* *..........* *...*G*......* *..........* *..........* *..........*

Consensus *g.........* *..g.......* *..........* *..........* *..........* *..........* *...a..t...* *..........* *..........* *..........*

4701 4800

AB719398 *GTATTAGATC* *TGAACCTGCC* *CACGTGGGAT* *GACAGCAAAT* *TGATATCCAT* *CATAC*G*CGAT* *GCAAAACCAG* *ATGTGGTGTT* *GGTGCACCAC* *AATGTCACAA*

PvEV-2-Tan *..........* *..........* *..........* *..........* *.*A*........* *.....A....* *..........* *..........* *..........* *..........*

PvEV-2-SEN *..*G*.......* *..........* *..........* *.........*C *..........* *.....A....* *..........* *..........* *..........* *..........*

Consensus *..a.......* *..........* *..........* *.........t* *.g........* *.....a....* *..........* *..........* *..........* *..........*

4801 4900

AB719398 *AACGAAGGAT* *TGGCAAA*T*TA* *CTAACTAC*T*A* *TCAAGGTCGA* *GACAATACAT* *TCTTACCAAT* *CAAAAGAGGC* *CAACGCAGTG* *TTAGT*G*GTGC* *AGTACAATGA*

PvEV-2-Tan *..........* *...*T*...C..* *........C.* *..........* *..........* *..........* *..........* *..........* *.....A....* *..........*

PvEV-2-SEN *..........* *.......C..* *........C.* *..........* *..........* *..........* *..........* *..........* *.....A....* *..........*

Consensus *..........* *...c...c..* *........c.* *..........* *..........* *..........* *..........* *..........* *.....a....* *..........*

4901 5000

AB719398 *GGGGGGCAAT* *TCCCAGATCT* *ATATGAACA*G *AAATTACGCG* *GTTTCCGCAG* *CAACTAGGTG* *CAAAACCAAG* *TTGGTGTGGG* *TAAGCGTTGG* *ATTGCCCGAC*

PvEV-2-Tan *..........* *..........* *.........A* *..........* *..........* *..........* *..........* *..........* *..........* *..........*

PvEV-2-SEN A*.........* *..........* *.*C*.......A* *..........* *..........* *..........* *..........* *..........* *..........* *..........*

Consensus *g.........* *..........* *.t.......a* *..........* *..........* *..........* *..........* *..........* *..........* *..........*

5001 5100

AB719398 *GGGATGAAAC* *TAGTAGACAA* *ACTAAAAGGG* *TCATCACTGG* *ATCACCGTGG* *CGAAGGTTTG* *CAAGACGCCA* *TTGAATTGAC* *TCCAAACACC* *AGGCATATAA*

PvEV-2-Tan *.........*T *..........* *...*G*......* *..........* *..........* *..........* *..........* *..........* *..........* *..........*

PvEV-2-SEN *..........* *..........* *..........* *..........* *.*C*........* *..........* *..........* *.......*A*..* *..........* *..........*

Consensus *.........c* *..........* *...a......* *..........* *.t........* *..........* *..........* *.......g..* *..........* *..........*

5101 5200

AB719398 *TACGCCAAGC* *AATTGACAGT* *GGTACAATTT* *CTCCAGCTAC* *CCTAACCATA* *TTACAGACTA* *CCCAGTTGAG* *GCGGGAACAG* *ATCGAAATCA* *AACCCGAAGA*

PvEV-2-Tan *..........* *..........* *..........* *..........* *..........* *..........* *..........* *..........* *..........* *..........*

PvEV-2-SEN *..........* *......*T*...* *..*C*.......* *..........* *...*G*......* *........*Y*.* *..........* *..........* *..........* *..........*

Consensus *..........* *......c...* *..t.......* *..........* *...a......* *........t.* *..........* *..........* *..........* *..........*

5201 5300

AB719398 *GCAGCGGATC* *GAACGAGACT* *T*G*TTCACAAT* *GGAAGATGTA* *GAGAAGTTAG* *TGCAGGAACT* *GGACATCTAC* *AATCAAAGAG* *TTTGGACACC* *CATGGATTGG*

PvEV-2-Tan *..........* *..........* *.A........* *..........* *..........* *..........* *..........* *..........* *..........* *..........*

PvEV-2-SEN *..........* *..........* *.A........* *..........* *........*G*.* *..........* *..........* *..........* *..........* *......*C*...*

Consensus *..........* *..........* *.a........* *..........* *........a.* *..........* *..........* *..........* *..........* *......t...*

5301 5400

AB719398 *GGGGAATTCA* *AAAATAGGTG* *GCGGGCATCT* *GTCCCACCAG* *GGACTAAACT* *GGAAGTGGAC* *GATGACCGGC* *GCGTCTTGAC* *AGCCAAAGTA* *TGGGGGTGCA*

PvEV-2-Tan *..*A*.......* *..........* *..........* *..*T*.......* *..........* *..........* *..........* *..........* *..........* *..........*

PvEV-2-SEN *..........* *..........* *.....*T*....* *..........* *..........* *..........* *..........* *..........* *.........*G *.....*A*....*

Consensus *..g.......* *..........* *.....c....* *..c.......* *..........* *..........* *..........* *..........* *.........a* *.....g....*

5401 5500

AB719398 *CAGTCACGGT* *GACAGTTCAG* *CAAGACATGT* *CAAAATTTGC* *AATGGACTTA* *CAAATATCGT* *TTCTGATGCG* *ACTTGCTAAA* *TCAAAAATCG* *AAAAACAATT*

PvEV-2-Tan *..........* *..........* *..........* *..........* *..........* *.....*G*....* *..........* *..........* *..........* *..........*

PvEV-2-SEN *..........* *..........* *..........* *........*A*.* *..........* *..........* *..........* *..........* *........*T*.* *..........*

Consensus *..........* *..........* *..........* *........g.* *..........* *.....a....* *..........* *..........* *........c.* *..........*

5501 5600

AB719398 *TAAACAACAT* *TATAAAGACC* *TCTGGATAAG* *CGAAGCAATG* *GGCAGAGACT* *GCAAGTCAAT* *CATCATGGAC* *CTAAGGCCAG* *AGTACAGGGA* *TGATTTTGCA*

PvEV-2-Tan *..........* *..........* *..........* *..........* *..........* *..........* *..........* *..........* *.........*G *..........*

PvEV-2-SEN *..........* *..........* *..........* *.*R*........* *..*T*.......* *..........* *..........* *..........* *..........* *..........*

Consensus *..........* *..........* *..........* *.g........* *..c.......* *..........* *..........* *..........* *.........a* *..........*

5601 5700

AB719398 *CGCATTGTAA* *GACCAGCGCT* *TATGACCAAC* *ATGGTTAGAC* *CAATGCCGGA* *CACACAACCG* *TCGACATCAA* *CTCTAAACAA* *AGACAAAGCA* *ATAGTGATAG*

PvEV-2-Tan *..........* *..........* *..........* *..........* *..........* *..........* *..........* *..........* *..........* *..........*

PvEV-2-SEN *..........* *..........* C*.........* *......*G*...* *....*A*.....* *..........* *.....*T*....* *..........* *........*TG G*....*A*....*

Consensus *..........* *..........* *t.........* *......a...* *....g.....* *..........* *.....a....* *..........* *........ca* *a....g....*

5701 5800

AB719398 *ATTGGCAAGT* *AACTGACGAT* *GAAAGTAGTG* *GTGAAACACA* *ATCAACCCCA* *CGGCACGCAG* *AGATTGAGGA* *CTGTGACGAT* *AACACACTAG* *GTGACGTGGA*

PvEV-2-Tan *..........* *..........* *..........* *..........* *..........* *..........* *.......*A*..* *........*G*.* *..........* *..........*

PvEV-2-SEN *..........* *..........* *..........* *...*R*......* *..........* *..........* *..........* *..........* *..........* *..........*

Consensus *..........* *..........* *..........* *...a......* *..........* *..........* *.......g..* *........a.* *..........* *..........*

5801 5900

AB719398 G*AGATCGGGG* *TTGATAGGAG* *CGTCGGACAT* *CAGATTCTTG* *TACCCAGTAA* *CAAACAATAT* *GACACCAAAG* *CAGGCATCAC* *TAATTGCAGA* *TGACACAAAC*

PvEV-2-Tan *A.........* *..........* *.*C*........* *..........* *..........* *..........* *..........* *..........* *..........* *..........*

PvEV-2-SEN *A.........* *..........* *..........* *..........* *..........* *..*G*.......* *..........* *........*C*.* *..........* *..........*

Consensus *a.........* *..........* *.g........* *..........* *..........* *..a.......* *..........* *........a.* *..........* *..........*

5901 6000

AB719398 *CCCCATTTGT* *TACCTGTTAA* *AAGCATTGAA* *TTGTTGGCGT* *TGGTACGAGC* *GGCATTGCCC* *ACAATAAGGT* *TAACCGTACT* *GTTCGAAGAA* *TGCGACAACG*

PvEV-2-Tan *..........* *..........* *..........* C*.........* *..........* *..........* *..........* *..........* *..........* C*.........*

PvEV-2-SEN *..........* *..........* *..........* *..........* *..........* *..........* *..........* *..........* *..........* *..........*

Consensus *..........* *..........* *..........* *t.........* *..........* *..........* *..........* *..........* *..........* *t.........*

6001 6100

AB719398 *GCTACAATCT* *AGATTTAGTG* *AGTGAGGAAA* *GCAAGGTGGC* *CATGTTT*A*AA* *GTAACGCAGC* *ACAACATGGT* *GGTTAGGTCA* *ATTGGGGGGA* *CAATGAAATT*

PvEV-2-Tan *..........* *..........* *..........* *..........* *.......G..* *..........* *..........* *..........* *..........* *..........*

PvEV-2-SEN *..........* *..........* *..........* *..........* *.......G..* *..........* *..........* *...*C*......* *..........* *..........*

Consensus *..........* *..........* *..........* *..........* *.......g..* *..........* *..........* *...t......* *..........* *..........*

6101 6200

AB719398 *GAGAATGGGG* *CAGTTACTCC* *TTGACGCAAA* *GTTGAAAGTA* *TTTGCCACGC* *AAA*T*AAAGCG* *TGGTGGTATT* *TGGAACCGTA* *GTCCAATGTT* *GACAGATGAA*

PvEV-2-Tan *..........* *..........* *..........* *..........* *..........* *...C......* *..........* *..........* *..........* *..........*

PvEV-2-SEN *..........* *..........* *..........* *..........* *......*G*...* *...C......* *..........* *.....*Y*....* *..........* *...*G*......*

Consensus *..........* *..........* *..........* *..........* *......a...* *...c......* *..........* *.....c....* *..........* *...a......*

6201 6300

AB719398 *CAAACCCAAA* *TCGTGTTAGC* *AGCATACCCC* *TGGATGCAAG* *TTTATCTGGA* *TCTGGAC*C*AA* *GCACAGGACG* *AAAATGTTCT* *AATCAAGCAC* *ATCAGTCAAC*

PvEV-2-Tan *..........* *..........* *..........* *..........* *..........* *.......A..* *..........* *..........* *.........*T *..........*

PvEV-2-SEN *..........* *..........* *..........* *........*R*.* *..........* *.......A..* *..........* *..........* *......*A*...* *..........*

Consensus *..........* *..........* *..........* *........a.* *..........* *.......a..* *..........* *..........* *......g..c* *..........*

6301 6400

AB719398 *TGGACAGCTA* *TGATTACACT* *GCCAGGGTGG* *CAGAGTTTAA* *AAACGTTTTG* *GCTAGATATG* *ACGAATTGTC* *TCAAATTTAC* *TCGCAAACAG* *GCGAATTGGT*

PvEV-2-Tan *..........* *..........* *..........* *..........* *......*C*...* *..........* *..........* *..........* *..........* *..........*

PvEV-2-SEN *..........* *..........* *..........* *....*A*.....* *..........* *..........* *..........* *..........* *..........* *..........*

Consensus *..........* *..........* *..........* *....g.....* *......t...* *..........* *..........* *..........* *..........* *..........*

6401 6500

AB719398 *TGATGTTAGC* *GGTGTTAAGT* *GCTGCGGGCC* *GCTTTTAAAT* *GAAAGCATGA* *TAGATGCATT* *CGGCAACGCA* *AACACGGTAA* *TGACCCTCAA* *CCTAGA*C*GTG*

PvEV-2-Tan *..........* *.....*C*....* *..........* *..........* *..........* *..........* *..........* *..........* *..........* *......T...*

PvEV-2-SEN *..........* *..........* *..........* *..........* *..........* C*.........* *..........* *..........* *..........* *......T...*

Consensus *..........* *.....t....* *..........* *..........* *..........* *t.........* *..........* *..........* *..........* *......t...*

6501 6600

AB719398 *GGGCCAACGT* *CCAACAGAAT* *AAT*A*AGATTA* *CTGAGTGAAT* *GGACAGAGAC* *ACAGTATGAC* *ATGGGTCAGA* *TGGC*T*AAGTT* *AGATGTTCT*G *GGGAGCAGTG*

PvEV-2-Tan *..........* *..........* *...T......* *..........* *.......*A*..* *..........* *..........* *....A.....* *.........*A *..........*

PvEV-2-SEN *..........* *.*T*........* *...T......* *..........* *..........* *..........* *..........* *....A...*C*.* *.........*C *..........*

Consensus *..........* *.c........* *...t......* *..........* *.......g..* *..........* *..........* *....a...t.* *.........*. *..........*

6601 6700

AB719398 *TTGTGATGGC* *GACTTACGGG* *GGTTGCACGT* *TATGCGCTGG* *AATTAAGTTT* *TCATCTCGTG* *GCGTAGATTT* *GATGAAAGTG* *GGCAGGCAAA* *GCACAGCAA*C

PvEV-2-Tan *..........* *..........* *..........* *..........* *..........* *..........* *..........* *..........* *..........* *.........T*

PvEV-2-SEN *..........* *..........* *..........* *..........* *..........* *..........* *..........* *..........* *..*T*.......* *.........T*

Consensus *..........* *..........* *..........* *..........* *..........* *..........* *..........* *..........* *..c.......* *.........t*

6701 6800

AB719398 *CAGCAATAGA* *TGCATTACTA* *TATACCACCA* *CCCGGACATG* *TCCGTGACGG* *CCGCTCTGTG* *TCACGGGTTA* *CACCTCAATG* *ACAAATT*G*AA* *CCTGGTGACC*

PvEV-2-Tan *..........* *..........* *..........* *..........* *..........* *.*T*........* *..........* *..........* *.......A..* *..........*

PvEV-2-SEN T*........*G *..........* *..........* *..........* *..........* *....*A*.....* *.........*G *..........* *.......A..* *..........*

Consensus *c........a* *..........* *..........* *..........* *..........* *.c..t.....* *.........a* *..........* *.......a..* *..........*

6801 6900

AB719398 *CTCGGACCTC* *TGGACTATCC* *ACCAGATCTG* *TT*G*GCAGATT* *TAGACGACAC* *CATGAAAGAA* *CCATGGTTTG* *AACTGGGTAT* *GATTTTTGAA* *AGGATGTCAC*

PvEV-2-Tan *..........* *..........* *..........* *..A.......* *..........* *..........* *..........* *..........* *..........* *..........*

PvEV-2-SEN *.........*Y *..........* *..........* *..A.....*A*.* *.*G*........* *..........* *..........* *..........* *..........* *..........*

Consensus *.........c* *..........* *..........* *..a.....t.* *.a........* *..........* *..........* *..........* *..........* *..........*

6901 7000

AB719398 *ACATGAATAA* *ATGGCTTGGC* *TCCAAGATGA* *CTGGTGTGGA* *TAGTGAACAC* *ACATGTGATT* *TGTACGGAGA* *CACGAATCAC* *GGGCTACTCA* *ATGACTTAGC*

PvEV-2-Tan *..........* *..........* *..........* *..........* *..........* *..........* *..........* *..........* *..........* *..........*

PvEV-2-SEN *..........* *.......*A*..* *..........* *..........* *..........* *..........* *..........* *......*C*...* *.........*G *.*Y*........*

Consensus *..........* *.......g..* *..........* *..........* *..........* *..........* *..........* *......t...* *.........a* *.t........*

7001 7100

AB719398 *AAAACAGCAA* *CCGAAATTGG* *AAATTCCGCT* *GCGTAATAGT* *GTCCCCACAC* *ACCGGAGCCT* *GTTGAATAGA* *CCATTCAAAG* *CCAT*A*ATAAA* *GGGCCACCAA*

PvEV-2-Tan *..........* *..........* *..........* *......*C*...* *..........* *..........* *..........* *..........* *....G.....* *..........*

PvEV-2-SEN *..........* *..........* *....*Y*.....* *..........* *..*T*.......* *..........* *...*A*......* *..........* *....G.....* *..........*

Consensus *..........* *..........* *....t.....* *......t...* *..c.......* *..........* *...g......* *..........* *....g.....* *..........*

7101 7200

AB719398 *GTGGTCATGG* *CGAGTTATGA* *GGGAATCATG* *TATTGCTCAA* *ACACTATGTG* *GGCCATGAGA* *ATGAGCATGA* *GGTTTATGCC* *ACTAGATGTT* *ATGGTACAAA*

PvEV-2-Tan *..........* *..........* *..........* *.....*T*....* *..........* *..........* *..........* *....*C*.....* *..........* *..........*

PvEV-2-SEN *..........* *..........* *..........* *..........* *..........* *..........* *..........* *..........* *..........* *..........*

Consensus *..........* *..........* *..........* *.....c....* *..........* *..........* *..........* *....t.....* *..........* *..........*

7201 7300

AB719398 *GGAGTTACAC* *ATACAAAGCC* *AACAGCTGGC* *CCTGGAGACT* *GGCAAGGTTC* *GTAACATTGA* *ACGGATCACT* *GGCTAACATG* *ATGGTGGGTT* *ACGACGATGA*

PvEV-2-Tan *..........* *..........* *..........* *..........* *.........*T *..........* *..........* *..........* *..........* *..........*

PvEV-2-SEN *..........* *..........* *..........* *..........* *..........* *..........* *..........* *..........* *..........* *..........*

Consensus *..........* *..........* *..........* *..........* *.........c* *..........* *..........* *..........* *..........* *..........*

7301 7400

AB719398 *AACACCAACA* *GGGGCCTTGA* *ATTTGCCACT* *AGAGTATCAC* *GAAAA*C*AACA* *AGACCATTGT* *GCATAGGTTT* *TTGTGCGAAA* *AGCGCGAAGA* *AGTGGT*G*TTG*

PvEV-2-Tan *..........* *..........* *..........* *..........* *.....T....* *..........* *..........* *..........* *..........* *......T...*

PvEV-2-SEN *..........* *..........* *..........* *...*A*..*C*...* *.....T....* *..........* *..........* *..........* *..........* *...*A*..T...*

Consensus *..........* *..........* *..........* *...g..t...* *.....t....* *..........* *..........* *..........* *..........* *...g..t...*

7401 7500

AB719398 *TCCACCATGA* *AGAAAACACA* *TCACGTGGTG* *TTCGTCAGCA* *GGAGTCAACT* *GCAGAATTAT* *GGGTCTATGA* *TCAGCCGGGA* *CTGCGCGGGT* *CTCCCAGTCG*

PvEV-2-Tan *..........* *..........* *..........* *..........* *..........* *..........* *..........* *..........* *..........* *..........*

PvEV-2-SEN *..........* *..........* *..........* *..........* *....*C*.....* A*.........* *..........* *.*T*........* T*..*T*..*A*...* *..........*

Consensus *..........* *..........* *..........* *..........* *....t.....* *g.........* *..........* *.c........* *c..c..g...* *..........*

7501 7600

AB719398 *AAGAACAGGG* *GTGTGACACA* *ATTGACCAAG* *GGTTTGATTT* *ATTGGTAGAA* *CA*G*ATGTGCC* *TAAAATACTA* *CTATAACTCA* *ATAGAAAAAG* *GTGAAGGGAG*

PvEV-2-Tan *..........* *..........* *..........* *..........* *..........* *..A.......* *..........* *..........* *..........* *..........*

PvEV-2-SEN *....*G*.....* *..........* *..........* *..........* *..........* *..A.......* *..........* *..........* *..........* *..........*

Consensus *....a.....* *..........* *..........* *..........* *..........* *..a.......* *..........* *..........* *..........* *..........*

7601 7700

AB719398 *TGTGATGGTG* *ACTGACTTTC* *CTTATTTATC* *ACTACTCACG* *TCAAGTTGGG* *GAAATTTCTG* *CATACCGGGA* *ACAAATGGCG* *GAGGATTTAG* G*TATTACCAA*

PvEV-2-Tan *..........* *..........* *..........* *..........* *..........* *..........* *..........* *..........* *..........* *A.........*

PvEV-2-SEN *..........* *..........* *..........* *..........* *..........* *..........* *..........* *........*T*.* *..........* *A.........*

Consensus *..........* *..........* *..........* *..........* *..........* *..........* *..........* *........c.* *..........* *a.........*

7701 7800

AB719398 *AATTTAGTGG* *ATTGCAACAA* *CATGATGGAA* *AAAATGAGTC* *ACGTGTACTG* *CATAGACGAG* *AGAGTTAATG* *AAGAAAACAA* *AGACCAAAGC* *GCATACTACA*

PvEV-2-Tan *..........* *..........* *..........* *..........* *..........* *..........* *..........* *..........* *..........* *..........*

PvEV-2-SEN *..........* *..........* *..........* *..........* *..........* *..........* *..........* *..........* *..........* *..........*

Consensus *..........* *..........* *..........* *..........* *..........* *..........* *..........* *..........* *..........* *..........*

7801 7900

AB719398 *AAGAGATGCA* *GGAGGCAATA* A*ACACCCAGA* *TAAGAACAGG* *CACCGGGCCT* *TGGTTCACCA* *ATAGCAACAG* *CAACGAACCA* *GCACGTATAA* *CACCAAAAAA*

PvEV-2-Tan *..........* *..........* *G.........* *..........* *..........* *..........* *..........* *..........* *..........* *..........*

PvEV-2-SEN *..........* *..........* *G....*T*....* *..........* *..........* *..........* *..........* *..........* *..........* *..........*

Consensus *..........* *..........* *g....c....* *..........* *..........* *..........* *..........* *..........* *..........* *..........*

7901 8000

AB719398 *CTTAATGGGA* *TTGGTGATTG* *CCAAATTGCC* *ATGTCAGGAG* *ATTGCGAGCA* *TGATGCGAGC* *CAACAACTTG* *ACTTCTATGA* *ATATAATACT* *GCCACAAAAT*

PvEV-2-Tan *..........* *..........* *..........* *..........* *..........* *..........* *..........* *..........* *..........* *..........*

PvEV-2-SEN *..........* *..........* *..........* *..........* *..........* *..........* *.........*A *.....*G*....* *..........* *.........*Y

Consensus *..........* *..........* *..........* *..........* *..........* *..........* *.........g* *.....t....* *..........* *.........t*

8001 8100

AB719398 *GTGCAGAGTG* *ACAATCAGTT* *ATTTGGTATA* *GCAACCGAAA* *CACGGCGGGA* *GTATCAGTTT* *TGGTACCACA* *ATTCGAACCA* *TTTGACAACA* *ATAAACAAGG*

PvEV-2-Tan *..........* *..........* *..........* *..........* *..........* *..........* *..........* *..........* *..........* *..........*

PvEV-2-SEN *..........* *....*C*.....* *..........* *..........* *..........* *..........* *..........* *..........* *..........* *..........*

Consensus *..........* *....t.....* *..........* *..........* *..........* *..........* *..........* *..........* *..........* *..........*

8101 8200

AB719398 *AGTTGTTGCA* *CATGTTTATG* *ACAGGGAAGT* *GCATTACAAC* *TAAGTATGGT* *ACCCTAGTCA* *GTCATGGAGC* *CAATCGAGTG* *CTTGGACATT* *GCATAGTAAA*

PvEV-2-Tan *....*A*.....* *..........* *..........* *..........* *..........* *..........* *..........* *..........* *..........* *..........*

PvEV-2-SEN *..........* *..........* *.....*A*....* *..........* *..........* *..........* *..........* *..........* *..........* *..........*

Consensus *....g.....* *..........* *.....g....* *..........* *..........* *..........* *..........* *..........* *..........* *..........*

8201 8300

AB719398 *ATTGACGTTG* *TTGCAAAATG* *CGGAAATGGC* *ACCCAA*A*TAT* *GTGCGCCCCA* *TAGTGCACGA* *CAACCAAAAG* *AAGCAGGTGG* *TATTCAAACT* *ACCCGTAATA*

PvEV-2-Tan *..........* *.........*T *..........* *......G...* *..........* *..........* *..........* *..........* *..........* *..........*

PvEV-2-SEN *..........* *..........* *.........*Y *......G...* *..........* *..........* *..........* *.....*A*....* *.*G*........* G*.........*

Consensus *..........* *.........g* *.........c* *......g...* *..........* *..........* *..........* *.....g....* *.a........* *a.........*

8301 8400

AB719398 *GCCAATTTTA* *AAATGTTTCT* *GAACGGTGGG* *GAGGCACTG*A *TTTACAAAGA* *AGTGGACATG* *TCATTAAAGA* *TGTATAGAGC* *ACTGTCACTC* *AGAATGTTGA*

PvEV-2-Tan *..........* *..........* *..........* *.........G* *..........* *..........* *..........* *..........* *..........* *..........*

PvEV-2-SEN *..........* *..........* *..........* *.........G* *..........* *..........* *..........* *....*C*.....* *..........* *..........*

Consensus *..........* *..........* *..........* *.........g* *..........* *..........* *..........* *....t.....* *..........* *..........*

8401 8500

AB719398 *GACCTGACAC* *AACAATAGAT* *GATTTGTTGT* *CCTATGCCAG* *AACTTACAGT* *CACACCGTCG* *CTTATTCAAT* *TAGCAAAATG* *ACAAGCCAGC* *AACCCCAAAT*

PvEV-2-Tan *..........* *..........* *..*C*.......* *..........* *..........* *..........* *..........* *..........* *..........* *..........*

PvEV-2-SEN *.*G*........* *..........* *..........* *..........* G*.........* *..........* *..........* *..........* *..........* *..........*

Consensus *.a........* *..........* *..t.......* *..........* *a.........* *..........* *..........* *..........* *..........* *..........*

8501 8600

AB719398 *GATAACAAAA* *TTGATGGAGT* *GTTGTGTGTG* *TGTCTTCTTA* *GAGAGCACTA* *GGTTGAACAA* *TGCCACTACA* *CGATTAACTG* *AGTTAATCAA* *CAGTACTGA*A

PvEV-2-Tan *.........*G *..........* *..........* *.........*G *..........* *..........* *..........* *.....*G*....* *..........* *.........G*

PvEV-2-SEN *..........* *..........* *..........* *..........* *..........* *.......*T*..* *......*C*...* *..........* *..........* *.........G*

Consensus *.........a* *..........* *..........* *.........a* *..........* *.......c..* *......t...* *.....a....* *..........* *.........g*

8601 8700

AB719398 *TTCCCACTCA* *CAAAACTGTC* *AGGACTCAAA* *GACATGGCCT* *GGTTTGCAAC* C*ATCAAAATG* *GTCACTGACG* *TTTATAAGTG* *GCTGGGTGTT* *GACGTCGGAA*

PvEV-2-Tan *..........* *..........* *..........* *..*T*.......* *..........* *T.........* *..........* *..........* *..........* *..........*

PvEV-2-SEN *..........* *..........* *..........* *..........* *..........* *T.........* *..........* *..........* *..........* *..........*

Consensus *..........* *..........* *..........* *..c.......* *..........* *t.........* *..........* *..........* *..........* *..........*

8701 8800

AB719398 *TTGAGCAGTT* *ACTTGAAATG* *GTGAGCACCG* *T*G*GCAAAGGA* *GGCAGCAGGC* *AGAATAATCA* *ACAACATTGA* *CAAATTGAAG* *GCAGTGAGAG* *TGAGCAAAGT*

PvEV-2-Tan *..........* *..........* *..........* *.A........* *..........* *..........* *..........* *..........* *..*T*.......* *..........*

PvEV-2-SEN *..........* *..........* *..........* *.A.....*A*..* *..........* *..........* *..........* *..........* *..........* *..........*

Consensus *..........* *..........* *..........* *.a.....g..* *..........* *..........* *..........* *..........* *..a.......* *..........*

8801 8900

AB719398 *GGAACACACC* *GACCCATTGA* *TTATGTATGA* *TGC*A*TCAGCC* *GATCAGACAG* *TAATATCGGA* *CCATGGGCCA* *TCCAAACCCG* *TACAATTCGT* *CCAGGGGGTA*

PvEV-2-Tan *..........* *..........* *..........* *...G......* *.....*A*....* *..........* *...*C*......* *..........* *..........* *..........*

PvEV-2-SEN *..........* *..........* *..........* *...G*G*.....* *..........* *..........* *......*A*...* A*....*G*....* *..........* Y*.....*AA*..*

Consensus *..........* *..........* *..........* *...gt.....* *.....g....* *..........* *...t..g...* *t....a....* *..........* *c.....gg..*

8901 9000

AB719398 *ATAAAGACTT* *TAACGCTCGG* *GAAAGCAGCT* *GGATATGGCA* *TGGCCAGATC* *AGCACAGAAC* *CTGCGAGATC* *CAAGAGTTGA* *TAACTTGGTG* *GCCAAACCAT*

PvEV-2-Tan *........*C*.* *..........* *..........* *..........* *..........* *..........* *..........* *..........* *..........* *.........*N

PvEV-2-SEN *..........* *...*A*......* *..........* *..........* *..........* *........*G*.* *.....*G*....* *..........* *.*G*........* *.*Y*........*

Consensus *........t.* *...c......* *..........* *..........* *..........* *........a.* *.....a....* *..........* *.a........* *.c.......t*

9001 9100

AB719398 *TGGCAATATC* *CAAAGTGGTC* *AAGCAGTTGG* *AAGAGCACAT* *AGGGGAG*G*AC* *AAGGCCGAAG* *CACTACGAGA* *GGTGATGCAG* *GATTTGCATT* *ATGATTACCC*

PvEV-2-Tan A*.........* *..........* *..........* *..........* *.......A..* *..........* *..........* *..........* *.....*A*....* *..........*

PvEV-2-SEN *..........* *..........* *..........* *..........* *.......A..* *..........* *.......*G*..* *..........* *...*C*......* *..........*

Consensus *t.........* *..........* *..........* *..........* *.......a..* *..........* *.......a..* *..........* *...t.g....* *..........*

9101 9200

AB719398 *CAATAGTCAA* *TTAATGGAGG* *CATTAATAAA* A*GTAATGGAG* *AACATAGATG* *A*C*GAACTGAA* *ATGGAGGGAT* *ATGTGTTATT* *TTGGCCAGAT* *CATGAGAGGT*

PvEV-2-Tan *..........* *..........* *..........* *G.........* *..........* *.T..*C*.....* *.........*C *..........* *..........* *..........*

PvEV-2-SEN *..........* C*.........* *..........* *G.........* *..........* *.T........* *..........* *..........* *.*C*..*T*.....* *..........*

Consensus *..........* *t.........* *..........* *g.........* *..........* *.t..a.....* *.........t* *..........* *.t..c.....* *..........*

9201 9300

AB719398 *GAATGGCACA* *CC*A*TTAGTGC* *AAATTTAGCC* *AAAAAATCAA* *AGAAGCCAGT* *CAACACATGC* *ATCACATTGA* *ACCAGTTAGA* *CAAAGAAATT* *AAAGAGTTGC*

PvEV-2-Tan *..........* *..G.......* *..........* *..........* *..........* *..........* *..........* *..........* *..........* *..........*

PvEV-2-SEN *..........* *..G.......* *..........* *..........* *..........* *..........* *..........* *..........* *..........* *..........*

Consensus *..........* *..g.......* *..........* *..........* *..........* *..........* *..........* *..........* *..........* *..........*

9301 9400

AB719398 *AATGGATGTA* *TCCAACTGAA* *TGCAACCATT* *GGAAAGGGAA* A*AAAATTGTA* *TTTAGTACAA* *TAGGCAGTAG* *AGGGGACATC* *GAACCTTACA* *TTGCGTGGGC*

PvEV-2-Tan *..........* *..........* *..........* *..........* *G.....*C*...* *..........* *..........* *..........* *..........* *..........*

PvEV-2-SEN *..........* *..........* *..........* *..........* *G.........* *..........* *..........* *..........* *..........* *..........*

Consensus *..........* *..........* *..........* *..........* *g.....t...* *..........* *..........* *..........* *..........* *..........*

9401 9500

AB719398 *ACAAGTTGTG* *GCCAAACTAG* *GGAGTGAATG* *CAAGTTTTTG* *GTGCCAAAAG* *ACTACGTTGA* *TTATGTGAAC* *AGCTACGGAT* *TCGAGGCATT* *GGGGTTGCAG*

PvEV-2-Tan *..........* *..........* *.*A*........* *..........* *..........* *..........* *..........* *..........* *..........* *..........*

PvEV-2-SEN *..........* T*.........* *..........* *..........* *..*A*.......* *..........* *..........* *..........* *..........* *..........*

Consensus *..........* *g.........* *.g........* *..........* *..g.......* *..........* *..........* *..........* *..........* *..........*

9501 9600

AB719398 *GTAGATTCTA* *GCAAATTAAT* *CAACAGCTGC* *ATACAAATGG* *AGAAGCACAA* *ATGGAACCCG* *CTACAACTCT* *ATAATGAATT* *GAATGAGATG* *TTTGAAATAA*

PvEV-2-Tan *..........* *..........* *......*T*...* *..........* *..........* *..........* *..........* *..........* *..........* *..........*

PvEV-2-SEN *..........* *.....*C*....* *..........* *..........* *..........* *..........* *..........* *..........* *...*Y*......* *..........*

Consensus *..........* *.....t....* *......c...* *..........* *..........* *..........* *..........* *..........* *...t......* *..........*

9601 9700

AB719398 *TAGAAGGCAT* *GTTCAAACTG* *AACACCACCA* *AACTCTTACA* *GTTTTGCGAA* *GGTGTTAACA* *TGATGATTGA* *GACACCATTC* *ACCCACGTTG* *GGGTGCAAGT*

PvEV-2-Tan *..........* *..........* *..........* *..........* *..........* *..........* *..........* *..........* *..........* *..........*

PvEV-2-SEN *..........* *.........*A *..........* *.......*G*..* *..........* *.....*A*....* *..........* A*........*T *........*G*.* *..........*

Consensus *..........* *.........g* *..........* *.......a..* *..........* *.....t....* *..........* *g........c* *........t.* *..........*

9701 9800

AB719398 *GGCACAAAAA* *CTGAGGGCGC* *CCTGCTTATT* *CAGTACAGCT* *TACCCCTGGG* *AACAGCAAGC* *CGGCATGACA* *ACAAGGGCAG* *ACAGCGCCAC* *TATAATAGAG*

PvEV-2-Tan *..........* *..........* *..........* *..........* *..........* *..........* *...*T*......* *..........* *..........* *..........*

PvEV-2-SEN *......*G*...* *..........* *.*T*........* *..........* *..........* *..........* *..........* *..........* *...*A*......* C*.....*T*...*

Consensus *......a...* *..........* *.c........* *..........* *..........* *..........* *...c......* *..........* *...g......* *t.....a...*

9801 9900

AB719398 *ATGTTAACGG* *GCATAGCGGC* *ATTTACCCCG* *TTTCGCAAGC* *ACATTGAACA* *ATGGCGTCAC* *AGAACATTGC* *AATTGCACAA* *TCCGCGGGGA* *ATAATGGCGC*

PvEV-2-Tan *..........* *..........* *..........* *..........* *..........* *..........* *..........* *..........* *..........* *..........*

PvEV-2-SEN *..........* *.*T*........* *..........* *..........* *..........* *..........* *..........* *..........* *..........* *..........*

Consensus *..........* *.c........* *..........* *..........* *..........* *..........* *..........* *..........* *..........* *..........*

9901 10000

AB719398 *ACGGCAGCGG* *TAACCCGCTG* *GTGTACTTAC* *ACCCTAAGTT* *AACCACATGG* *TGGAAAACAT* *CCAACACCTC* *AGCGTGCGTG* *GGTTATGCAA* *ACAGTTGTGT*

PvEV-2-Tan *..........* *..........* *..........* *..........* *..........* *..........* *..........* *..........* *..........* *..........*

PvEV-2-SEN *.....*NNNN*.* C*.........* *......*C*...* *..........* *..........* *..........* *..........* *...*T*......* *..........* *..........*

Consensus *.....agcg.* *t.........* *......t...* *..........* *..........* *..........* *..........* *...g......* *..........* *..........*

10001 10100

AB719398 *AAAGCAAGTC* *AGCGAGCAAG* *ATGTGGAGCT* *AACTAAATGG* *GCAGCAGAAG* *CCAAAACAAT* *TGCAGTGTGT* *TTTGGATCAA* *TGACGGGGGA* *TCGCAGAAGC*

PvEV-2-Tan T*.........* *..........* *..........* G*.........* *..........* *..........* *......*A*...* *..........* *........*A*.* *...*T*......*

PvEV-2-SEN *..........* *..........* *....*A*.....* *..........* *..........* *..........* *..........* *..........* *..........* *..........*

Consensus *a.........* *..........* *....g.....* *a.........* *..........* *..........* *......g...* *..........* *........g.* *...c......*

10101 10200

AB719398 *AACATGACAA* *CCAAATTAAT* *GTTATCTATG* *CAATCACAAT* *ATAGATTTTT* *AGTAGTGGAT* *GGGCAGACCA* *GTTATGA*C*CA* *AATTAAATTT* *CCAAACATGA*

PvEV-2-Tan *..........* *..........* *..........* *..........* *..........* *..........* *..........* *.......T..* *..........* *..........*

PvEV-2-SEN *.*G*........* *.......*G*..* *..........* *..........* *........*G*.* *..........* *..........* *.......T..* *..........* *..........*

Consensus *.a........* *.......a..* *..........* *..........* *........t.* *..........* *..........* *.......t..* *..........* *..........*

10201 10300

AB719398 *GGCAGGT*G*TC* *TAGTTGCAAT* *TACAATCTAT* *TGTTCAACGC* *GGTGGAAAT*T *GTGGTCACCC* *ATGGCGGCAG* *TGGCACCACG* *CACAATGCAT* *TGCAGCACGG*

PvEV-2-Tan *.*A*.....A..* *...*C*......* *..........* *..........* *.........C* *..........* *..........* *..........* *..........* *..........*

PvEV-2-SEN *.......A..* *..........* *........*G*.* *..........* *.........C* *..........* *..........* NNNNN*.....* *..........* *..........*

Consensus *.g.....a..* *...t......* *........a.* *..........* *.........c* *..........* *..........* *tggca.....* *..........* *..........*

10301 10400

AB719398 *CTGCGGTGTG* *ATAATTGACC* *CCCATTTTGG* *CGACCAATTC* *GCTTGGCTAA* *AATCAGTGGA* *ACAGCTTGGC* *TGTGGAACTA* *GCTTAGAAAA* *ACTTCTGAAA*

PvEV-2-Tan *..........* G*.........* *..........* *..........* *..........* *..........* *..........* *..........* *..........* *......*A*...*

PvEV-2-SEN *..........* *..........* *..........* *......*G*...* *..........* *..........* *..........* *.......*TC*.* *....*G*.....* *..........*

Consensus *..........* *a.........* *..........* *......a...* *..........* *..........* *..........* *.......ct.* *....a.....* *......g...*

10401 10500

AB719398 *TTGGACGTCA* *ATGA*C*CAAAT* *AA*T*TAAACTG* *CAACAGTACA* *CTGACACAGC* *CCAAACTCTG* *GGTGGGACAG* *TACGCGCCGA* *GAACTTCATG* *GTAAACCTAA*

PvEV-2-Tan *..........* *.*C*..A.....* *..G.......* *..........* *..........* *..........* *.....*A*....* *..........* *..........* *..........*

PvEV-2-SEN *......*A*...* *....A.....* *..G.......* *..........* *..........* *..........* *..........* *.*G*........* *..........* *..........*

Consensus *......g...* *.t..a.....* *..g.......* *..........* *..........* *..........* *.....g....* *.a........* *..........* *..........*

10501 10600

AB719398 *TCACAAACAG* *TGAAGTGCTG* *TGGCAGGCGG* *CGTCCGACCA* *CACCACCAAT* *TTGCTCGCCA* *ACGTGGTCGA* *ACCACTGGT*T *AGCTTAGATT* *TAAATTGTAA*

PvEV-2-Tan *..........* *..*G*.......* *..........* *..........* *..........* *..........* *..........* *.........*C *..........* *..........*

PvEV-2-SEN *..........* *..........* *.....*TTNNN NN*........* *..........* *..*A*.......* *....*A*.....* G*........*A *..........* *..........*

Consensus *..........* *..a.......* *.....ggcgg* *cg........* *..........* *..g.......* *....g.....* *a........*. *..........* *..........*

10601 10700

AB719398 *AAACATCAAC* *AACCAACTGG* *CAAGCGGCGG* *TTTGGGTAAG* *TGGGCAGACG* *TGGAAGACAC* *AACCGTGATC* *ATCACAGCAG* *AAGGGCCCAA* *ATTTAACAAA*

PvEV-2-Tan *..........* *..........* *..........* *..........* *..........* *..........* *..........* *..........* *..........* *....*G*.....*

PvEV-2-SEN *..........* *..........* *........*A*.* *..........* *..........* *..........* *.........*Y *.........*A *.*T*........* *........*G*.*

Consensus *..........* *..........* *........g.* *..........* *..........* *..........* *.........c* *.........g* *.a........* *....a...a.*

10701 10800

AB719398 *AATCACATTG* *AACGGTCCAA* *CCAAATGAAG* *AAAGGCACAG* *GTCCTCAGCA* *CAATCAAGGT* *TCGAGCACGA* *CTGTACACCC* *ACAGACAGGA* *AGGAATCCAG*

PvEV-2-Tan *..........* *..........* *..........* *..........* *..........* *........*A*.* *.......*T*..* *..........* *.......*A*..* *..........*

PvEV-2-SEN R*.........* G*.........* *..........* *..........* *........*G*.* *..........* *..........* *..........* *..........* *......*T*...*

Consensus *a.........* *a.........* *..........* *..........* *........c.* *........g.* *.......c..* *..........* *.......g..* *......c...*

10801 10900

AB719398 *AGATAGGAAG* *GAACTATAAA* *GAGTACCAAT* *CATCACATGC* *ACTAACATAC* *GTGCCT*G*CCA* *ATGATGACTT* *GCAATTGGCT* *GCCAAAGCGC* *GAGAGGAAAG*

PvEV-2-Tan *...*C*......* *..........* *..........* *..........* *..........* *......A...* *.*C*........* *..........* *..........* *..........*

PvEV-2-SEN *....*G*.....* *..........* *..........* *..........* *......*G*...* *......A*NT*.* *...*G*......* *..........* *..........* *..........*

Consensus *...ta.....* *..........* *..........* *..........* *......a...* *......acc.* *.t.a......* *..........* *..........* *..........*

10901 11000

AB719398 *AAAAGAACAA* *AAAAAACAGG* *CCGACGCCAC* *AAGACGCAAG* *AT*C*GCAGTGC* *AGCTAGCCGA* *GGACCGGAAT* *AGGAGAAAAG* *AAGCCAACGA* *AGAAAAGAAG*

PvEV-2-Tan *..........* *.....*C*....* *..........* *..........* *..T.......* *.*T*........* *..........* *..........* *..........* *..........*

PvEV-2-SEN *.........*G G*......*T*..* *..*A*.......* *..........* *..T.......* *......*Y*...* *..........* *.....*GG*...* *.....*G*.*A*..* *.........*A

Consensus *.........a* *a....a.a..* *..g.......* *..........* *..t.......* *.g....c...* *..........* *.....aa...* *.....a.c..* *.........g*

11001 11100

AB719398 *AAAGCAACGC* *AACTACGTCT* *CGAACAAAGT* *GAACTTAGCA* *AAGAACTGAA* *GGCAGCAGCA* *TCGTTAG*T*GA* *TTGAAACCAA* *CCCAGAAGGG* *AAGCCG*A*ATG*

PvEV-2-Tan *..........* *..........* *..........* *..........* *..........* *..........* *.......C..* *..........* *..........* *.....*A*G.*NN

PvEV-2-SEN *...*A*......* *..........* *..........* *.....*C*.*A*..* *........*C*.* *......*C*...* NNNNN*..C*A*.* *..........* *..........* *......G...*

Consensus *...g......* *..........* *..........* *.....t.g..* *........a.* *......a...* *tcgtt..cg.* *..........* *..........* *.....gg.tg*

11101 11200

AB719398 *AAA*C*A*T*ATAC* *ACTCAGTA*T*A* *AAAT*T*GAAAG* *AACCAAAGTA* *TCTATGGGGA* *ATGCCAAAAG* *ACGAAGACGC* *AAGAGAGAAA* *GAGATGA*T*CA* *GTGAATGCGC*

PvEV-2-Tan NNNNNN*....* *........*NG C*...C.....* *.*G*........* C*.........* *..........* *..........* *..........* *.......C..* *..........*

PvEV-2-SEN *...*T*.*C*....* *........*G*.* *....C.*G*...* *..........* *..........* *..........* *..........* *..........* *.......C..* *.....*C*....*

Consensus *aaa*.*a*.*....* *........*.*a* *a...c.a...* *.a........* *t.........* *..........* *..........* *..........* *.......c..* *.....t....*

11201 11300

AB719398 *GGTA*G*CAGCG* *ATCACGGAAC* *CACCAGCAGC* *GGTTTACGTA* *ACAACAACGG* *GCACAACAAT* *TGAACCTGAG* *AGTGTGGAAT* *CCCAAGGGGA* *GCGACAAGCG*

PvEV-2-Tan *....A.....* *........*G*.* *.........*A *..........* *..........* *..........* *..........* *..........* *..........* *..........*

PvEV-2-SEN *..*NN*A.....* *.*C*........* *..........* *....*C*.....* *..........* *..........* *.*A*........* *..........* *........*A*.* *..........*

Consensus *..taa.....* *.t......a.* *.........c* *....t.....* *..........* *..........* *.g........* *..........* *........g.* *..........*

11301 11400

AB719398 *GAGCCATTGA* *GCAAGGCACA* *CGAACAATGC* *GGAACTATGG* *ATTCAGAAAA* *GCTGTACACC* *AGGACCAACC* *TCATCTATGA* *CTTGTTTGAA* *GTGGAGCACA*

PvEV-2-Tan *..........* *..........* *..........* *.....*C*....* *..........* *..........* *..........* *..........* *..........* *..........*

PvEV-2-SEN *..........* *..........* *..........* *..........* *..........* *..........* *..........* *..........* *..........* *..........*

Consensus *..........* *..........* *..........* *.....t....* *..........* *..........* *..........* *..........* *..........* *..........*

11401 11500

AB719398 *CCGGAACAAA* *TTATGCACCA* *TTCAAAATCA* *TGTCACCAAC* *AGGGCAATCG* *GTAGTATTAA* *ATCCCAATGA* *GGGAACGGAT* *TGCGTTTTTG* *AATGTCTCAG*

PvEV-2-Tan A*.........* *.........*G *..........* *..........* *..........* *..........* *..........* *..........* *..........* *..........*

PvEV-2-SEN *..........* *..........* *..........* *..........* *.*A*........* *........*G*.* *..........* *..........* *.....*C*....* *..........*

Consensus *c.........* *.........a* *..........* *..........* *.g........* *........a.* *..........* *..........* *.....t....* *..........*

11501 11600

AB719398 *AGTGGCACTG* *ACAGATGAGT* *ATACAAATGA* *TGAAGACAAA* *TCAACCATTA* *TCCTCAGAAC* *CTGTTATCAA* *TGGATGAACA* *TTCAAACCAT* *GCCAACCACC*

PvEV-2-Tan *..........* *..........* *..........* *..........* *..........* *..........* *..........* *..........* *..........* *...*G*......*

PvEV-2-SEN *..........* *..........* *..........* *..........* *........*C*.* *..........* *..........* *..........* *..........* *..........*

Consensus *..........* *..........* *..........* *..........* *........t.* *..........* *..........* *..........* *..........* *...a......*

11601 11700

AB719398 *CGCCAGTTAG* *AGGGGTTGGT* *GAGAGCATTA* *CATTTGCACA* *TTGGAGTCCA* *GTTACCCAGT* *TGCAGCATCG* *ATTTCCCAAT* *CGGGAGTGCA* *ACCATTGAGC*

PvEV-2-Tan *..........* *..........* *..........* *..........* *.*C*........* *..........* *..........* *..........* *....*T*.....* *..........*

PvEV-2-SEN *..*T*.......* *..........* *..........* *........*T*.* *..........* *..........* *..........* *..........* *..........* *..........*

Consensus *..c.......* *..........* *..........* *........c.* *.t........* *..........* *..........* *..........* *....a.....* *..........*

11701 11800

AB719398 *AAGTCACTTA* *TTTGGAGATC* *ATAGAGGGAG* *TGCTCGCTGC* *ACACTGTGTT* *TTAAAGAAAG* *TTTCGAAGCC* *AAGTGTGGAG* *TTCTACACAG* *CGTCAAATGC*

PvEV-2-Tan *..........* *..........* *..........* *..........* *..........* *..........* *..........* *..........* *..........* *....*G*.....*

PvEV-2-SEN *..........* *..........* *..........* *..........* *..........* *..*G*..*A*....* *..........* *..........* *..........* *..*G*.......*

Consensus *..........* *..........* *..........* *..........* *..........* *..a..g....* *..........* *..........* *..........* *..t.a.....*

11801 11900

AB719398 *CCCCCAAATG* *TCATTTTCAG* *TTAATGTAAT* *GAATGC*T*GAA* *AATGCTGCAA* *TAGACGATGG* *GTTAGAACCA* *AAATTGACCA* *TGTCCAGCTG* *GGCACTGGAC*

PvEV-2-Tan *..........* *..........* *..........* *......C...* *..........* *..........* *..........* *.*G*........* *..........* *..........*

PvEV-2-SEN *......*G*...* *..........* *.......*G*..* *......C...* *..........* *..........* *......*G*...* *..........* *..........* *..........*

Consensus *......a...* *..........* *.......a..* *......c...* *..........* *..........* *......a...* *.a........* *..........* *..........*

11901 12000

AB719398 *TTGTT*C*AATG* *ACAGTAAATG* *TGAGAGATGG* *TGGGCTATGA* *TGCGCACCAA* *CTATACTTAT* *GCGGAATTCA* *TGCACAAACT* *GGAATCAAAA* *AACCTAGTCC*

PvEV-2-Tan *.....T....* *..........* *..........* *..........* *..........* *...*C*......* *.....*G*....* *..........* *..........* *.....*G*....*

PvEV-2-SEN *..*A*..T*G*...* *..........* *..........* *..........* *..........* *..........* *..........* *..........* A*.........* *..........*

Consensus *..g..ta...* *..........* *..........* *..........* *..........* *...t......* *.....a....* *..........* *g.........* *.....a....*

12001 12100

AB719398 *ACACTGCCAA* *ACGCATGCAT* *GCAGCCATGT* *CTGT*G*ACTAA* T*TGTACCCAG* *TCAGAAAATA* *TCTTAACAGT* *TGATGAAAAA* *GTAGTGGCGA* *ACAAATGGTA*

PvEV-2-Tan *..........* *..........* T*.........* *....A.....* C*.........* *..........* *..........* *..........* *..........* *..........*

PvEV-2-SEN *..........* *..........* *..........* *....A.....* Y*..*C*......* *..........* *..........* *..........* *...*A*......* *..........*

Consensus *..........* *..........* *g.........* *....a.....* .*..t......* *..........* *..........* *..........* *...g......* *..........*

12101 12200

AB719398 *TTGGGTAACG* *GACGGAACCA* *AGTGGTATGG* *GGCAATATCC* *AGACGGGTGA* *ATGAAAGGAG* *TTTATTGATA* *ACAACAGCCT* *CACATCATAA* *ATGGGGCGAA*

PvEV-2-Tan C*.........* *..........* *..........* *..........* *..........* *..........* *..........* *..........* *..........* *..........*

PvEV-2-SEN *..........* *......*G*...* *..........* A*.........* *..........* *....*M*.....* *..........* *.........*C *....*G*.....* *..........*

Consensus *t.........* *......a...* *..........* *g.........* *..........* *....a.....* *..........* *.........t* *....t.....* *..........*

12201 12300

AB719398 *TTGGTTGTAA* *TGGTGCGGAC* *GGATAAC*T*AC* *TTGGGTTGGC* *CAACTGGACC* *AACTGTGAGA* *GAGCGGTTG*A *CACGCAAGAT* *TATAGTGCCC* *GGGAATCAAG*

PvEV-2-Tan *........*C*.* *..........* *.......C..* *..........* *..........* *..........* *..*A*......G* *.*G*........* *..........* *..........*

PvEV-2-SEN *.....*G*....* *..........* *.......C..* *..........* *...*T*......* *..........* *.........G* *..........* *..........* *..........*

Consensus *.....t..a.* *..........* *.......c..* *..........* *...c......* *..........* *..g......g* *.a........* *..........* *..........*

12301 12400

AB719398 *ATGTCACGGC* *GCTAAACAAG* *TCCACATTGT* *ACGAAAGCAG* *GTGCAAAAAT* *TACGGGGTCC* *AATTAAAAGG* *GGTGCAAGAC* *ACCAGCTGTG* *A*A*TATGTAGC*

PvEV-2-Tan *..........* *..........* *..........* *..........* *..........* *.....*A*....* *..........* *..........* *..........* *.G........*

PvEV-2-SEN *..........* *.*T*........* *..........* *.......*T*..* *..........* *......*A*...* *....*G*.....* *...*R*......* *..........* *.G........*

Consensus *..........* *.c........* *..........* *.......c..* *..........* *.....gg...* *....a.....* *...g......* *..........* *.g........*

12401 12500

AB719398 *AGTGTTTGAC* *TATGATAACC* *GGTCACACCA* *TA*G*GTACGAC* *GACGCTGCCT* *TTTTGGTCAG* T*AAGGGTCCA* *AGTAAGAGCA* *TCGGATTGGG* *TTTCGACCTG*

PvEV-2-Tan *..........* *..........* *..........* *..A.......* *..........* *..........* *C.........* *..........* *..........* *..........*

PvEV-2-SEN *..........* *..........* *..........* *..A.......* *..........* *..........* *C.........* *..........* *..........* *...*T*......*

Consensus *..........* *..........* *..........* *..a.......* *..........* *..........* *c.........* *..........* *..........* *...c......*

12501 12600

AB719398 *ACCGGAGATA* *TGTGGGCCAA* *AATAACTAAC* *CGCACAAGTG* *CCGTCAGGCA* *CATGCTGAAC* *AGAGGAAAAC* *TGACATTGTG* *TATGGAGTTT* *GTAGACCACC*

PvEV-2-Tan *..........* *..........* *..........* *..........* *..........* *..........* *.....*G*....* *..........* *..........* *..*G*.......*

PvEV-2-SEN *........*A*.* *..........* *.........*T *..........* *..........* *..........* *..........* *..........* *..........* *..........*

Consensus *........t.* *..........* *.........c* *..........* *..........* *..........* *.....a....* *..........* *..........* *..a.......*

12601 12700

AB719398 *AACAACGAAA* *TACATTTGCA* *AGCGTTCTTG* *ACGCTAGTAG* *A*A*TAAAATA*T *ACTTTGGAAG* *GTGGCAGTGT* *TTATTTGGCA* *ACGGAAGAAG* *TGTCGCATGC*

PvEV-2-Tan *..........* *..........* *..........* *..........* *.G.......C* *..........* *..........* *......*A*...* *..........* *..........*

PvEV-2-SEN *..........* *......*C*...* *..........* *..........* *.G.......C* *..........* *..........* *..........* *..........* *..........*

Consensus *..........* *......t...* *..........* *..........* *.g.......c* *..........* *..........* *......g...* *..........* *..........*

12701 12800

AB719398 *ACACTCAAAT* *TTGATACAAG* *AGTGGGCTGT* *GTATATGAAT* *GCCAGCAACG* *GATTAGTGAT* *CCCAGATAGA* *ACATTAATGG* *AAAGATTAAA* *AGTGGGAGTA*

PvEV-2-Tan *..........* *..........* *..........* *..........* *..........* *..........* T*.........* *..........* *..........* *..........*

PvEV-2-SEN *..........* *..*A*.......* *..........* *..........* *....*A*.....* *....*G*..*A*..* *..........* *..........* *...*A*......* *..........*

Consensus *..........* *..g.......* *..........* *..........* *....g.....* *....a..g..* *c.........* *..........* *...g......* *..........*

12801 12900

AB719398 *CCATTGCAAG* *AGTTAGTGGA* T*CAAATTTGC* *CCAGACATCA* *ACATCAAGTC* *AGCATTCGGT* *AAAAGGCACC* *CTAGCAAACA* *CACAGCGGTG* *TTGACTAATG*

PvEV-2-Tan *..........* *..........* *C.........* *..........* *..........* *..........* *..........* *..........* *...*G*......* *..........*

PvEV-2-SEN *........*C*.* *..........* *C.........* *..........* *..........* *..........* *..........* *..........* *..........* *..........*

Consensus *........a.* *..........* *c.........* *..........* *..........* *..........* *..........* *..........* *...a......* *..........*

12901 13000

AB719398 *ACCATGCAAG* *GGT*A*AAAATG* *CCGTTGACAA* *CAATAGTGAA* *AACAGAAATG* *GAA*A*CCCAAG* *ACAAATATTT* *AGTGTTGAGG* *TCCAACTGCA* *TCATTTGTGA*

PvEV-2-Tan *..........* *...G......* *..........* *..........* G*.........* *...G......* *..........* *..........* *..........* *..........*

PvEV-2-SEN *......*A*...* *...G......* *..........* *..........* *..........* *...G......* *..........* *......*A*...* *..........* *..........*

Consensus *......c...* *...g......* *..........* *..........* *a.........* *...g......* *..........* *......g...* *..........* *..........*

13001 13100

AB719398 *ATTACTCCCA* *CTAACTAGAG* *GTGGTGGAGA* *AGAAGAACCC* *CTTTT*T*TGGA* *GACACGGGGA* *AGAATTAAAA* *GTGAACTCTG* *TGA*T*CCGCTG* *GTGGAATCAA*

PvEV-2-Tan *..........* *..........* *.......*T*..* *..........* *.....C....* *..........* *..........* *..........* *...C......* *..........*

PvEV-2-SEN *...*R*......* *..........* *.*C*........* *..........* *.....C....* *.......*A*..* *..........* *..*R*.......* *...C......* *..........*

Consensus *...a......* *..........* *.t.....a..* *..........* *.....c....* *.......g..* *..........* *..g.......* *...c......* *..........*

13101 13200

AB719398 *CCGGAAACCG* *AGGCGCCCAA* *AACTGGATTC* *AATAAAGATA* *AATATTTGCA* *AATCAATAGC* *GAAGCTTTAA* *CAAACTGGAA* *CGCAGAAAAT* *ACATCAGTGG*

PvEV-2-Tan *..........* *..........* *..........* *..........* *..........* *..........* *..........* *..........* *........*G*.* *..........*

PvEV-2-SEN *..........* *.......*T*..* *..........* *..........* *.....*C*....* *..........* *..........* *..........* *..........* G*.........*

Consensus *..........* *.......c..* *..........* *..........* *.....t....* *..........* *..........* *..........* *........a.* *a.........*

13201 13300

AB719398 *TGCAAATTAG* *CACCAATCCA* *TTTAACCAAG* *CTGGGGATAG* *CACAAAAATG* *CACTATGTGG* *GAGTAACACA* *ATGGGCAAAT* *GTCGCTGACT* *CACAATTGGG*

PvEV-2-Tan *..........* *..........* *..........* *..........* *..........* *..........* *..........* *..........* *..........* *..........*

PvEV-2-SEN *..........* *..........* *........*T*.* *..........* *..........* *..........* *..........* *..........* *....*T*...*Y*.* *..........*

Consensus *..........* *..........* *........a.* *..........* *..........* *..........* *..........* *..........* *....c...c.* *..........*

13301 13400

AB719398 *TTCACCAGAG* *ATAGTAGACT* *ATGACATAAT* *GAGCATGTGG* *GATGACACCG* *ACTTTACGGA* *TTGGAATGAA* *CGATTTGCAC* *CGAAGAACGA* *CGTGGTTATT*

PvEV-2-Tan *..........* *..........* *..........* *..........* *..........* *..........* *..........* *..........* *..........* *......*C*...*

PvEV-2-SEN *..........* *.....*W*....* *..........* *..........* *..........* *....*C*.....* *..........* *..........* *..........* *.........*C

Consensus *..........* *.....a....* *..........* *..........* *..........* *....t.....* *..........* *..........* *..........* *......t..t*

13401 13500

AB719398 *AAATCAAAAG* *CCACAGCAAC* *AGAAATGCGA* *GTAACAACAA* *AATATACAAT* *GGTAGAGTAT* *CCAACCCACA* *GTAGACCAGT* *TTTGACTAAG* *GCGGCAAATC*

PvEV-2-Tan *..........* *..........* *..........* *..*G*.......* *..........* *..........* *..........* *..........* *..........* *..........*

PvEV-2-SEN *..........* *..........* *..........* *..........* *..........* *..........* *..........* *....*G*.....* *......*C*...* *..........*

Consensus *..........* *..........* *..........* *..a.......* *..........* *..........* *..........* *....a.....* *......t...* *..........*

13501 13600

AB719398 *AAGAGTTCAA* *TGCTGTTAGT* *GG*G*AGACT*A*C* *ACAACATAAC* *CACATACAGG* *TTAAAAAAAT* *ACGATCTTAC* *AAGAGAAGTG* *CGCAAATTTG* *TAGACATGTA*

PvEV-2-Tan *..........* *..........* *..*A*.....G.* *..........* *..........* *..........* *..........* *..........* *..........* *..........*

PvEV-2-SEN *..........* *..........* *..*R*.....G.* *..........* *..........* *..........* *....*C*.....* *..........* *........*C*.* *..........*

Consensus *..........* *..........* *..*.*.....g.* *..........* *..........* *..........* *....t.....* *..........* *........t.* *..........*

13601 13700

AB719398 *CTTTGATAGT* *TCAAAACGTG* *ACAAGTTGAA* *GGTTTATCAA* *ACTCAGCAAT* *TAACATTTAA* *TGACGCAAAA* *GTGCTAGATT* *GGTTAAGGGA* *CCGTCCGGAC*

PvEV-2-Tan *..........* *..........* *..........* *..........* *..........* *.......*C*..* *..........* *..........* *..........* *..........*

PvEV-2-SEN *..........* *........*C*.* *..........* *..........* *..........* *....*G*.....* *..........* *..........* *..........* *..........*

Consensus *..........* *........t.* *..........* *..........* *..........* *....a..t..* *..........* *..........* *..........* *..........*

13701 13800

AB719398 *AGTAATAAAA* *TAGCAGAAGA* *ACTGAACAAT* *ATTTTAGCTG* *AGGGTATGCA* *ACTGCACCCA* *AT*T*AATAAAT* *TAAACGTGCA* *CTTGAAATTG* *GAGTCGCTGT*

PvEV-2-Tan *.....*C*....* *..........* *..........* *..........* *..........* *..........* *..C.......* *..........* *...*A*......* *..........*

PvEV-2-SEN *..........* *..........* *..........* *..........* *..........* *..........* *..C..*C*....* *..........* *..........* *..........*

Consensus *.....t....* *..........* *..........* *..........* *..........* *..........* *..c..t....* *..........* *...g......* *..........*

13801 13900

AB719398 *TGAAATCAGA* *GCCAGCGTCA* *AATTTGAAAC* *AAGTCAAGGC* *TAGAGCTTTA* *GTTTGGCAGT* *GTAAAGGATA* *TTGCGCCATA* *TTTAGTCACG* *TGTTCAAAGA*

PvEV-2-Tan *..........* *..........* *..........* *..........* *..........* *..........* *.*C*........* *..........* *..........* *.*A*........*

PvEV-2-SEN *..........* *..........* *..........* *..........* *..........* *..........* *..........* *..........* *..*C*.......* *..........*

Consensus *..........* *..........* *..........* *..........* *..........* *..........* *.t........* *..........* *..t.......* *.g........*

13901 14000

AB719398 *AGTTAAAGTT* *AGACTCAAAG* *ACTTGTTGCG* *ACCAGAAATT* *GTGTATTCAG* *ATGGCTTAAG* *AGCGGATGAA* *TTGGCTGCAA* *GAGTGAGGTT* *GACCACAAAT*

PvEV-2-Tan G*.........* *..........* *..........* *..........* *..........* *..........* *..........* C*.........* *..........* *..........*

PvEV-2-SEN *..........* *..........* *.*T*........* *..........* *..........* *..........* *..........* *..........* *........*C*.* *..........*

Consensus *a.........* *..........* *.c........* *..........* *..........* *..........* *..........* *t.........* *........t.* *..........*

14001 14100

AB719398 *GTCAAATTTT* *TACTGGAAAA* *TGACTTGGCC* *CAACAAGACA* *AACAAACCGA* *TCATGAGATA* *ATCAAGGT*T*G* *A*G*ATGGAAAT* *TTATAAATTG* *TTAGGCGTGT*

PvEV-2-Tan *..........* *..........* *..........* *..........* *..........* *..........* *........C.* *.A........* *..........* *..........*

PvEV-2-SEN *..........* *.*G*........* *...*TC*.....* *.....*G*....* *..........* *..........* *........C.* *.A........* *..........* *.....*T*....*

Consensus *..........* *.a........* *...ct.....* *.....a....* *..........* *..........* *........c.* *.a........* *..........* *.....c....*

14101 14200

AB719398 *GTCC*C*CACCT* *AGT*G*GATTTA* *TGGAAAAATT* *GTCACCACAG* *TTGGGCCTAC* *AAGTCTAGAA* *CTGTAGCAGG* *TGTAGGTGAC* *GCTATGCGAC* *TCACTGGCCA*

PvEV-2-Tan *....T.....* *...A.....*G *..........* *..........* *..........* *..........* *..........* *..........* *..........* *..........*

PvEV-2-SEN *....T.....* *...A......* *..........* *..........* *..........* *..........* *....*G*.....* *..........* *..........* *..........*

Consensus *....t.....* *...a.....a* *..........* *..........* *..........* *..........* *....a.....* *..........* *..........* *..........*

14201 14300

AB719398 *AGCCACCACT* *GCTATAGGGA* *ACGCCATCAC* *CAATATGCTG* *GTGCATAGGC* *GGTTGGTTAA* *AACATTGGGT* *CACAACCTAA* *GATTGTTTTT* *GGTGTTGGGT*

PvEV-2-Tan *..........* *..........* *....*A*.....* *..........* *..........* *..........* *.*G*........* *..........* *....*A*.....* *..........*

PvEV-2-SEN *..........* *..........* *.*T*........* *...*Y*......* *..........* *..........* *..........* *..........* *..........* *..........*

Consensus *..........* *..........* *.c..c.....* *...t......* *..........* *..........* *.a........* *..........* *....g.....* *..........*

14301 14400

AB719398 *GATGATGGAT* *TAATGTTCAC* *AACAACATGG* *GTGGATACCA* *CTAAGTTGAA* *TAACGAGCTG* *AAAACCAATC* *ATAACATGAT* *GTGCAAACCA* *AGTTTAAGTC*

PvEV-2-Tan *..........* *.*G*........* *..........* *..........* *..........* *..........* *.....*T*....* *..........* *..........* *........*C*.*

PvEV-2-SEN *.....*C*....* *..........* *..........* *..........* *..........* *......*A*...* *..........* *..........* *..........* *..........*

Consensus *.....t....* *.a........* *..........* *..........* *..........* *......g...* *.....c....* *..........* *..........* *........t.*

14401 14500

AB719398 *AGACCCATGG* *TGTGTTTTGC* *TGCATGATGG* *CAGCAATAAC* *TGATGAAGGT* *AATTGCACAC* *TGGGACCAGA* *TGTGGTGCGA* *TTAAGACGCA* *GATTCGAGTG*

PvEV-2-Tan *..........* *..........* *..........* *..........* *..........* *..........* *..........* *..........* *..........* *..........*

PvEV-2-SEN *..........* *..........* *..........* *..........* *.........*C *..........* *..........* *..........* *..........* *..........*

Consensus *..........* *..........* *..........* *..........* *.........t* *..........* *..........* *..........* *..........* *..........*

14501 14600

AB719398 *TCCAAATGGG* *GTGAGTGAAA* *TGACTCCCGA* *CAATGTACAA* *GCGAGATGCA* *TGGCGTATTA* T*ATGATGCTC* *GGGGCTACAC* *CTGAAGTGAC* *ACGAGCAATA*

PvEV-2-Tan *..........* *..........* *..........* *..........* *..........* *..........* *C.........* *.....*C*....* *..........* *..........*

PvEV-2-SEN *..........* *..........* *..........* *..........* *..........* *..........* *C.........* *..........* *.......*A*..* *..........*

Consensus *..........* *..........* *..........* *..........* *..........* *..........* *c.........* *.....t....* *.......g..* *..........*

14601 14700

AB719398 *CAGCAACAAC* *AGTTGCCCAT* *ACAACCAATT* *AAATGGTATG* *ATGCGGACAG* *TATACGTAGT* *GCAACTATGA* *GTAAATACAA* *CATGGATGCC* *AACCAAGTTT*

PvEV-2-Tan *..........* *..........* *..........* *..........* *..........* *..........* *..........* *..........* *..........* *..........*

PvEV-2-SEN *..........* *..........* *..........* *..........* *..........* C*.........* *.....*C*....* *..........* *..........* *..........*

Consensus *..........* *..........* *..........* *..........* *..........* *t.........* *.....t....* *..........* *..........* *..........*

14701 14800

AB719398 *TGAACGAAGA* *GAGATTGTTG* *CTAGGCATGT* *TGAGTAAACC* *CAACAGCTTT* *GCACACAAAA* *TACTACATTG* *GCACGAAGGT* *CAA****TAG****CTTG* *AATTTTCGGG*

PvEV-2-Tan *..........* *..........* *..........* *..........* *......*T*...* *..........* *..........* *..........* *..........* *..........*

PvEV-2-SEN *..........* *......*A*...* *..........* *.....*G*....* *..........* *..........* *..........* *..........* *..........* *..........*

Consensus *..........* *......g...* *..........* *.....a....* *......c...* *..........* *..........* *..........* *..........* *..........*

14801 14820

AB719398 *GGGTGGGGGC* *CCCC*CCCCCC

PvEV-2-Tan *..........* *....*NNNNNN

PvEV-2-SEN *..........* *..*NNNNNNNN

Consensus *..........* *..cc*......
